# A cross-sectional study of inflammatory markers as determinants of circulating kynurenines in the Lung Cancer Cohort Consortium

**DOI:** 10.1038/s41598-023-28135-9

**Published:** 2023-01-18

**Authors:** Øivind Midttun, Arve Ulvik, Klaus Meyer, Hana Zahed, Graham G. Giles, Jonas Manjer, Malte Sandsveden, Arnulf Langhammer, Elin Pettersen Sørgjerd, Annelie F. Behndig, Mikael Johansson, Neal D. Freedman, Wen-Yi Huang, Chu Chen, Ross Prentice, Victoria L. Stevens, Ying Wang, Loïc Le Marchand, Stephanie J. Weinstein, Qiuyin Cai, Alan A. Arslan, Yu Chen, Xiao-Ou Shu, Wei Zheng, Jian-Min Yuan, Woon-Puay Koh, Kala Visvanathan, Howard D. Sesso, Xuehong Zhang, J. Michael Gaziano, Anouar Fanidi, Hilary A. Robbins, Paul Brennan, Mattias Johansson, Per M. Ueland

**Affiliations:** 1 Bevital AS, Laboratory Building, Jonas Lies Veg 87, 5021 Bergen, Norway; 2 Genomic Epidemiology Branch, International Agency for Research on Cancer, Lyon, France; 3 Cancer Epidemiology Division, Cancer Council Victoria, Melbourne, Australia; 4 Centre for Epidemiology and Biostatistics, School of Population and Global Health, The University of Melbourne, Melbourne, Australia; 5 Precision Medicine, School of Clinical Sciences at Monash Health, Monash University, Melbourne, Australia; 6 Department of Surgery, Skane University Hospital, Malmö, Sweden; 7 Lund University, Malmö, Sweden; 8 Department of Clinical Sciences Malmo, Lund University, Malmö, Sweden; 9 Department of Public Health and Nursing, Hunt Research Centre, Norwegian University of Science and Technology, Levanger, Norway; 10 Levanger Hospital, Nord-Trøndelag Hospital Trust, Levanger, Norway; 11 Department of Endocrinology, St. Olavs Hospital, Trondheim University Hospital, Levanger, Norway; 12 Department of Public Health and Clinical Medicine, Umea University, Umeå, Sweden; 13 Department of Radiation Sciences, Oncology, Umea University, Umeå, Sweden; 14 Metabolic Epidemiology Branch, Division of Cancer Epidemiology and Genetics, National Cancer Institute, Bethesda, MD USA; 15 Public Health Sciences Division, Fred Hutchinson Cancer Center, Seattle, USA; 16 American Cancer Society, Atlanta, USA; 17 University of Hawai’i Cancer Center, University of Hawaiʻi at Mānoa, Honolulu, USA; 18 Vanderbilt University Medical Center, Nashville, USA; 19 Department of Obstetrics and Gynecology, NYU Langone Health, New York, NY USA; 20 Department of Population Health, NYU Langone Health, New York, NY USA; 21 Perlmutter Comprehensive Cancer Center, NYU Langone Health, New York, NY USA; 22 University of Pittsburgh and UPMC Hillman Cancer Center, Pittsburgh, USA; 23 Healthy Longevity Translational Research Programme, Yong Loo Lin School of Medicine, National University of Singapore, Singapore, Singapore; 24 Johns Hopkins Institute for Clinical and Translational Research, Baltimore, USA; 25 Brigham and Women’s Hospital, Harvard Medical School, Boston, USA; 26 Harvard T.H. Chan School of Public Health, Boston, USA; 27 Brigham and Women’s Hospital, Boston, USA; 28 VA Boston Healthcare System, Boston, MA USA; 29 Université Claude Bernard Lyon 1, Lyon, France

**Keywords:** Biomarkers, Inflammation

## Abstract

Circulating concentrations of metabolites (collectively called kynurenines) in the kynurenine pathway of tryptophan metabolism increase during inflammation, particularly in response to interferon-gamma (IFN-γ). Neopterin and the kynurenine/tryptophan ratio (KTR) are IFN-γ induced inflammatory markers, and together with C-reactive protein (CRP) and kynurenines they are associated with various diseases, but comprehensive data on the strength of associations of inflammatory markers with circulating concentrations of kynurenines are lacking. We measured circulating concentrations of neopterin, CRP, tryptophan and seven kynurenines in 5314 controls from 20 cohorts in the Lung Cancer Cohort Consortium (LC3). The associations of neopterin, KTR and CRP with kynurenines were investigated using regression models. In mixed models, one standard deviation (SD) higher KTR was associated with a 0.46 SD higher quinolinic acid (QA), and 0.31 SD higher 3-hydroxykynurenine (HK). One SD higher neopterin was associated with 0.48, 0.44, 0.36 and 0.28 SD higher KTR, QA, kynurenine and HK, respectively. KTR and neopterin respectively explained 24.1% and 16.7% of the variation in QA, and 11.4% and 7.5% of HK. CRP was only weakly associated with kynurenines in regression models. In summary, QA was the metabolite that was most strongly associated with the inflammatory markers. In general, the inflammatory markers were most strongly related to metabolites located along the tryptophan–NAD axis, which may support suggestions of increased production of NAD from tryptophan during inflammation.

## Introduction

**Figure 1:**
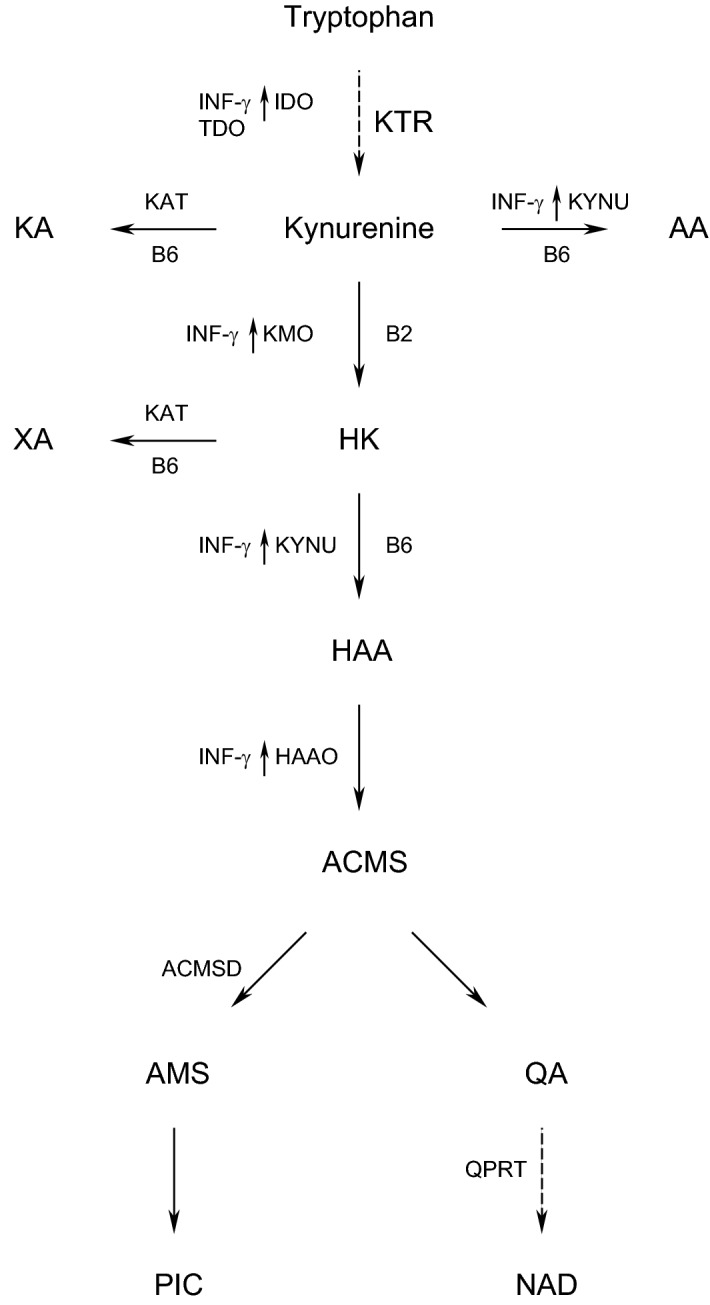
The kynurenine pathway of tryptophan metabolism, with enzymes and cofactors involved. Dashed arrow shafts indicate reactions with more than one step: Black arrows pointing up-wards indicate the enzymes which are stimulated by IFN-γ. AA: anthranilic acid; ACMS: α-amino-β-carboxymuconate-ε-semialdehyde; ACMSD: α-amino-β-carboxymuconate-ε-semialdehyde decarboxylase; AMS: α-aminomuconate-ε-semialdehyde; B2: vitamin B2 (flavin adenine dinucleotide); B6: vitamin B6 (pyridoxal 5′-phosphate, PLP); HAA: 3-hydroxyanthranilic acid; HAAO: 3-Hydroxyanthranilate 3,4-dioxygenase; HK: 3-hydroxykynurenine; IDO: indoleamine (2,3)-dioxygenase; KA: kynurenic acid; KAT: kynurenine aminotransferase; KMO: kynurenine 3-monooxygenase; KYNU: kynureninase; NAD: nicotine adenine dinucleotide; TDO: tryptophan (2,3)-dioxygenase; PIC: picolinic acid; QA: quinolinic acid; QPRT: quinolinate phosphoribosyltransferase; XA: xanthurenic acid.

The main part of the metabolism of tryptophan, an essential amino acid, takes place through the kynurenine pathway^1^, forming kynurenine and a number of downstream metabolites collectively called kynurenines (Fig. 1). A stimulator of cellular immune activation, the proinflammatory cytokine interferon-gamma (IFN-γ), whose activity is increased in inflammation, increases the production of neopterin by macrophages and also upregulates the enzyme indoleamine 2,3-dioxygenase (IDO), thereby increasing the conversion of tryptophan to kynurenine^1–3^ and concentrations of circulating kynurenine. Tryptophan may also be converted to kynurenine by tryptophan 2,3-dioxygenase (TDO)^1^, which is confined to the liver and not related to immune response and essentially does not affect the concentrations of circulating kynurenines^4^. Thus, increased levels of the kynurenine/tryptophan ratio (KTR) are determined by IDO activity^1,3^, and both KTR and neopterin are established IFN-γ induced inflammatory markers^5^. Kynurenine can be further converted to kynurenic acid (KA) by the vitamin B6 (pyridoxal 5'-phosphate, PLP) dependent enzyme kynurenine aminotransferase, to anthranilic acid (AA) by kynureninase, which is also vitamin B6 dependent, or to 3-hydroxykynurenine (HK) by the vitamin B2 (flavin adenine dinucleotide) dependent enzyme kynurenine mono-oxygenase. HK may be further converted to xanthurenic acid (XA) by kynurenine aminotransferase or to 3-hydroxyanthranilic acid (HAA) by kynureninase. Further metabolism of HAA by 3-hydroxyanthranilate 3,4-dioxygenase (HAAO) then forms α-amino-β-carboxymuconate-ε-semialdehyde (ACMS), which is either non-enzymatically converted to quinolinic acid (QA), or to picolinic acid. Further, QA may be further converted by quinolinate phosphoribosyl transferase (QPRT), leading to the de-novo formation of NAD^6,7^. It is considered that the main tissue responsible for this synthesis of NAD is the liver^6,7^, releasing nicotinamide into the blood and supporting NAD formation in other tissues^7^. It has been found that generation of NAD through the kynurenine pathway also takes place in macrophages, and regulates their immune function in inflammation^8^. Kynurenine mono-oxygenase^9–12^, kynureninase^9–11^ and HAAO^11^ are all upregulated by IFN-γ, and it has been shown that inflammation and immune response are associated with increased circulating concentrations of downstream metabolites in the kynurenine pathway^2,5,8,12–14^, and particularly QA levels are increased after activation by IFN-γ^15^.

A number of large prospective studies have investigated relations of circulating levels of KTR, kynurenines and neopterin with various diseases. Positive associations with all-cause mortality were found for KTR^16,17^, neopterin^16,17^, HK^16,17^, AA^16,17^ and QA^16^, while tryptophan^16,17^ was inversely associated and picolinic acid^16^ was not associated with this outcome. HAA, kynurenine, KA and XA have shown inconsistent associations with all-cause mortality^16,17^. Studies have also demonstrated positive associations of KTR^17–19^, kynurenine^17,20^, KA^21^, AA^21^, HK^17,20,21^ and HAA^21^, and inverse associations of tryptophan^17^ and XA^17^ with risk of cardiovascular disease, QA was not available in any of these studies. Positive associations of KTR^17,22^ and inverse associations of tryptophan^17^ and XA^17^ with overall cancer risk, and positive associations of KTR^23,24^ and QA^23^ with risk of lung cancer have also been reported. Where investigated, studies have demonstrated positive risks of similar strength for neopterin^17–19^ and C-reactive protein (CRP)^17^, an established non-specific marker of systemic inflammation^25^, with cardiovascular disease. Neopterin and CRP have also been reported to be positively associated with overall risk of cancer ^22^, as well as lung cancer^23,26,27^. Dietary quality showed qualitatively similar associations with plasma concentrations of CRP, neopterin and several kynurenines^28^.

Investigations of the associations of markers of inflammation with circulating kynurenines have focused only on correlations of kynurenines with neopterin^16,29^, KTR^16,29^ and CRP^16,17^. Only one of these works included QA, which was the kynurenine that was most strongly related with all-cause mortality^16^. To the best of our knowledge, only one paper has used regression models to investigate the association of markers of inflammation with circulating kynurenines^30^, and that work did not include QA. Given the common associations of established inflammation markers and kynurenines with disease, and the role of IFN-γ in upregulating several enzymes in the kynurenine pathway (Fig. 1), we therefore used different regression models on a large dataset to quantitatively investigate how circulating levels of neopterin and KTR (as measures that are closely related with IFN-γ activity), and CRP, performed as predictors of QA and six other kynurenines. We investigated both the magnitude of predictive power as well as potential nonlinear relations.

## Methods

### Study population

The Lung Cancer Cohort Consortium (LC3), which comprises 20 cohorts from the US, Nordic region, Asia and Australia, included 5364 case-controls pairs. For each case, one healthy, cancer-free control subject was matched by cohort, sex, race (US cohorts only), date of blood collection, date of birth, and self-reported smoking status. This work used data from the control subjects. The consortium has been described in detail previously^31^, as have the circulating concentrations of the biomarkers (except neopterin)^32^. Biomarker concentrations were missing for 50 subjects, leaving data from 5314 subjects available for this work. Demographic data for the total study population are shown in Table 1, and in detail for each geographic region and cohort in a previous publication^32^. All participants gave written, informed consent. The research was approved by the institutional review board of the International Agency for Research of Cancer.

**Table 1:** Characteristics of participants.

n	5364
n (US)	2400
n (nordic)	835
n (Asia)	1775
n (Australia)	354
Age, years^a^	62 (47, 75)
Male (%)	2456 (45.8)
BMI, kg/m^2a^	24.7 (19.3, 33.4)
Never smokers, n (%)^a^	1327 (24.7)
Former smokers, n (%)^a^	1518 (28.3)
Current smokers, n (%)^a^	2519 (47.0)
KTR, nmol/µmol	22.6 (16.4, 34.5)
Neopterin, nmol/L	11.0 (5.14, 23.6)
CRP, mg/L	1.77 (0.24, 13.2)
Tryptophan, µmol/L	66.5 (46.2, 90.7)
Kynurenine, µmol/L	1.51 (1.05, 2.22)
KA, nmol/L	43.9 (22.0, 91.2)
AA, nmol/L	13.9 (7.99, 29.1)
HK, nmol/L	36.6 (20.3, 70.6)
XA, nmol/L	12.4 (4.6, 29.1)
HAA, nmol/L	32.6 (16.1, 59.5)
QA, nmol/L	353 (208, 685)
Creatinine, µmol/L	74.5 (52, 105)
Cotinine, nmol/L	14.4 (1, 1870)
Riboflavin, nmol/L	19.2 (5.85, 89.6)
PLP, nmol/L	37.1 (13.8, 196)

Values are median (95% CI) or n (%). AA: anthranilic acid; BMI: body mass index; CRP: C-reactive protein; HAA: 3-hydroxyanthranilic acid; HK: 3-hydroxykynurenine; KA: kynurenic acid; KTR: kynurenine/tryptophan; PLP: pyridoxal 5′-phosphate; QA: quinolinic acid; XA: xanthurenic acid.

^a^Age, BMI and self-reported smoking status at date of blood draw.

### Blood samples and biochemical analyses

Serum/plasma was collected during the period 1974–2010, and all biospecimens were stored at ≤ − 80 °C until analysis, which was performed during 2012–2014. The samples were shipped on dry ice to Bevital AS (http://www.bevital.no) in Bergen, Norway, where all biochemical analyses were performed.

Briefly, circulating concentrations of tryptophan and kynurenine were obtained by mixing serum/plasma with ethanol to precipitate the proteins, and the supernatant was processed further before analysis by gas chromatography–mass spectrometry (GC–MS)^33^ using a Thermo Finnigan trace GC ultra system coupled to a Fisons MD800 mass spectrometer. The chromatographic column was a CP Sil 24-CB low-bleed/MS capillary column from Varian [15 m × 0.25 mm; film thickness 0.25 µm] and helium was used as carrier gas.

Analysis of KA, AA, HK, XA, HAA, neopterin (measured as total neopterin, i.e. the sum of neopterin plus 7,8-dihydroneopterin), vitamin B2 (riboflavin), vitamin B6 (pyridoxal 5'-phosphate, PLP) and cotinine (a marker of recent nicotine exposure^34^) were performed by precipitating the proteins using trichloroacetic acid. The supernatant was then injected onto a Zorbax stable-bond C8 reversed-phase column (150 × 4.6 mm, particle size 3.5 µm) equipped with a similar guard column (12.5 × 4.6 mm, particle size 5 µm) and the gradient mobile phase contained acetic acid, heptafluorobutyric acid and acetonitrile. High performance liquid chromatography–tandem mass spectrometry (LC–MS/MS)^35^ was performed using an Agilent series 1100 HPLC system coupled to an API 4000 mass spectrometer from Applied Biosystems. QA was included in this assay^35^ with the precursor-ion/product-ion 168.0/78.0 for the analyte and 171.0/81.0 for the internal standard (^2^H_3_-quinolinic acid). The limit of detection for quinolinic acid was 1.6 nmol/L.

Serum/plasma creatinine^36^ (often used as a marker of renal fuction) was quantified by precipitating proteins using trichloroacetic acid and injecting the supernatant onto a Fortis Phenyl column (150 × 4.6 mm; particle size, 3 μm) guarded by a Phenomenex Polar-RP SecurityGuard Cartridge (4 × 3.0 mm). The gradient mobile phase contained methanol and acetic acid. The LC–MS/MS system consisted of an Agilent series 1100 HPLC with an API 3000 mass spectrometer from Applied Biosystems.

Quantification of CRP was performed using miniaturized C18 columns (ZipTips) serving as the solid phase for antibody immobilization. Following column preparation, the ZipTips were incubated with serum samples and washed with PBS and water. The purified proteins were eluted from the column, and analysed by matrix-assisted laser desorption/ionization time of flight (MALDI-TOF) with a Bruker UltraFlextreme instrument^37^.

The CV for the analysis of neopterin was 12%, and 2.5–14.6% for the remaining biomarkers^32^. The kynurenine-to-tryptophan ratio (KTR) was calculated as kynurenine (nmol/L) divided by tryptophan (µmol/L).

### Demographic and lifestyle data at date of blood draw

BMI was calculated as weight/height^2^ (kg/m^2^), and cigarette smoking status classified as never, former and current smokers based on questionnaire responses by the participants. Smoking intensity was also assessed by circulating cotinine concentrations.

### Statistical methods

Discrete variables are given as counts (%). Continuous variables, including biomarkers (which were generally not normally distributed, and were therefore log-transformed before used in calculations of participant characteristics and correlations), are given as median with 95% CI. Circulating concentrations of cotinine lower than the limit of detection (1 nmol/L) were set to 1 nmol/L. For use in regression models, all continuous variables were further standardized by centering on the mean and dividing by the standard deviation. Thus, the model coefficients represents the mean difference in the log value of a given biomarker (in standard-deviation units) associated with a 1-unit difference in the independent variable.

Partial Spearman correlations were calculated using adjustment for cohort, age, sex, BMI, creatinine and cotinine, since these are all known to be related with circulating concentrations of kynurenines^29,32,38^. The correlations were also adjusted for sample storage time.

Mixed models of circulating levels of tryptophan, kynurenines and KTR were calculated by including cohorts and regions as random effects, and age, sex, BMI, CRP, creatinine, cotinine, riboflavin, PLP, sample storage time, and either KTR or neopterin, as fixed effects. Mixed models were also calculated for age groups below and above the median age, and for smoking categories (never, former and current smokers).

The relative importance of predictors of tryptophan, kynurenines and KTR were investigated by the lmg metrics (R^2^ partitioned by averaging over orders) using the relaimpo package for R^39^. Briefly, the algorithm evaluates all possible multiple linear regression models and all sequences for addition to a regression model that can be applied to a given set of predictors (regressors). The percentage explained variance of each regressor is then estimated as the average of these models. The models included cohort, age, sex, BMI, KTR or neopterin, CRP, creatinine, cotinine, riboflavin, PLP and sample storage time. These calculations also provided estimates of the total percentage of the variation in the outcomes that were explained by the models. These calculations were also performed for age groups below and above the median age, and for smoking categories (never, former and current smokers).

Nonlinear relations between continuous variables were investigated using generalized additive models (GAM) for tryptophan, kynurenines and KTR as outcomes, and cohort, age, sex, BMI, KTR or neopterin, CRP, creatinine, cotinine, riboflavin, PLP and sample storage time as predictors. We tested for a non-zero difference in slope of a segmented linear relationship by regressing tryptophan, kynurenines and KTR on neopterin, KTR and CRP. These segmented relations were fitted by segmented regression using the breakpoint value from Davies’ test as the starting estimate for the breakpoint between regression segments. The segmented regression models were adjusted for cohort, age, sex, BMI, and concentrations of cotinine, creatinine, riboflavin, PLP, CRP (included in models with adjustment for either neopterin or KTR) and sample storage time, and returned slopes of both segments, plus estimated breakpoint with 95% CI. Davies' test and segmented regression were also performed for age groups below and above the median age, and for smoking categories (never, former and current smokers). The GAMs and segmented regression models also provided estimates of the total percentage of variation explained by the models for each outcome. The p-values for the Davies' tests were adjusted by the method of Benjamini and Hochberg.

In all regression models, KTR and neopterin were included in separate models. KTR was included only in models of outcomes other than tryptophan, kynurenine and KTR. Models that contained KTR or neopterin gave similar regression coefficients for predictors other than KTR and neopterin, and for predictors other than KTR only results from models that included neopterin are therefore presented.

R version 4.0.3 for Windows (http://www.r-project.org) was used for all statistical calculations using the packages "lme4" for mixed models, "relaimpo" for the assessment of relative importance of predictors using multiple linear regression, "mgcv" for GAMs and "segmented" for segmented regression.

### Ethical approval

The protocol of the Lung Cancer Cohort Consortium was approved by the Ethics Committee of the International Agency for Research on Cancer (project number 11-13). The recruitment, data collection, and follow-up of the participating cohorts was approved by local institutional review boards. This study involved no additional contact or intervention with participants. This research was performed in accordance with the Declaration of Helsinki.

## Results

### Population characteristics

2400 of the participants were from the US, 835 from the Nordic region, 1775 from Asia, and 354 from Australia. The median (95% CI) age of the participants was 62 (47, 75) years, 45.8% were male, median BMI was 24.7 (19.3, 33.4) kg/m^2^, 24.7% were never, 28.3% former and 47.0% current smokers at the time of blood sampling (Table 1).The median age in age groups below and above the median were 55 and 68 years, respectively.

### Correlations including KTR, neopterin and CRP

**Figure 2:**
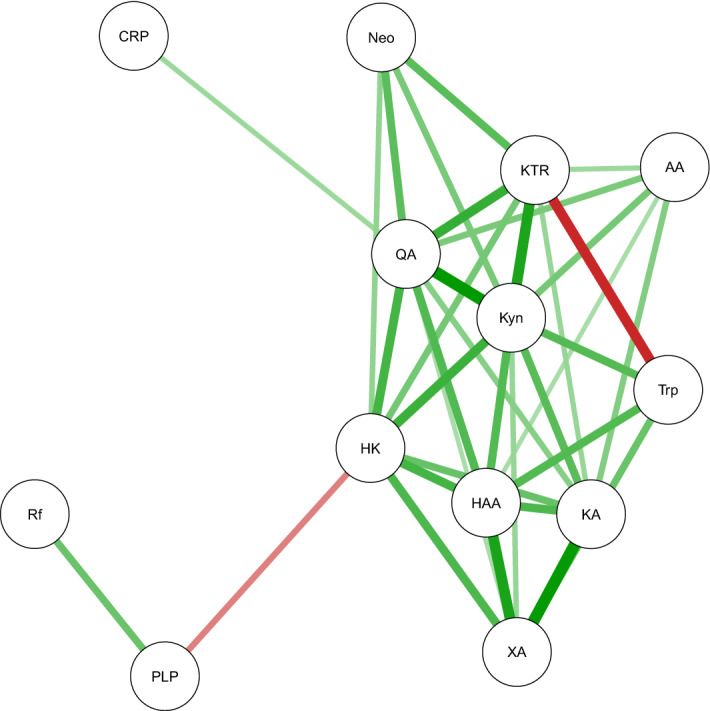
Qgraph plot of spearman correlations (including only absolute values > 0.2 to avoid a crowded figure) adjusted for cohort, sex, age, BMI, creatinine, cotinine and sample storage time. Green lines show positive correlations, red lines show inverse correlations. The color saturation and thickness of lines are proportional to the strength of the correlations. The figure illustrates the data given in Supplementary Table S2. AA: anthranilic acid; CRP: C-reactive protein; HAA: 3-hydroxyanthranilic acid; HK: 3-hydroxykynurenine; KA: kynurenic acid; KTR: kynurenine/tryptophan ratio; Kyn: kynurenine; Neo: neopterin; PLP: pyridoxal 5′-phosphate; Rf: riboflavin; Trp: tryptophan; QA: quinolinic acid; XA: xanthurenic acid.

The Qgraph plot in Fig. 2 based on multiply adjusted correlations (Supplementary Table S1) shows that KTR, neopterin, kynurenine and QA are located close together, with correlations in the range 0.31–0.52. CRP was located near these markers, and among the kynurenines CRP was most strongly correlated with QA (r = 0.23), while the correlations of CRP with KTR, neopterin and kynurenine were weaker (0.13 < r < 0.16). For the other kynurenines, KTR and neopterin were most strongly correlated with HK (r = 0.32 and 0.25, respectively), KA (r = 0.24 and 0.13) and AA (r = 0.23 and 0.17).

### KTR, neopterin and CRP as predictors of kynurenines and KTR in mixed models

In the mixed models, 1SD higher KTR was associated with 0.46 (95% CI 0.44 to 0.49) SD higher QA and 0.31 (95% CI 0.28 to 0.33) SD higher HK (Table 2). One SD higher neopterin was associated with 0.48 (95% CI 0.45 to 0.51) SD higher KTR and 0.44 (95% CI 0.41 to 0.46), 0.36 (95% CI 0.33 to 0.38) and 0.28 (95% CI 0.25 to 0.31) SD higher QA, kynurenine and HK, respectively. One SD higher CRP was associated with 0.14 (95% CI 0.12 to 0.17) SD higher QA and 0.11 (95% CI 0.08 to 0.13) SD higher HAA. These models gave essentially the same results within age groups (below and above the median) and smoking categories (Supplementary Tables S2 and S3, respectively).

**Table 2:** Predictors of kynureniners and KTR by mixed models.

	KTR	Tryptophan	Kynurenine	KA	AA	HK	XA	HAA	QA
Age	0.12 (0.09,0.14)	− 0.03 (− 0.06,0.0004)	0.10 (0.07,0.12)	0.05 (0.024,0.08)	0.09 (0.06,0.12)	0.006 (− 0.02,0.03)	− 0.05 (− 0.08,− 0.02)	− 0.09 (− 0.12,− 0.06)	0.10 (0.08,0.12)
Sex	0.35 (0.28,0.41)	− 0.44 (− 0.52,− 0.36)	− 0.02 (− 0.08,0.04)	0.09 (0.02,0.16)	− 0.0003 (− 0.06,0.06)	0.29 (0.22,0.36)	0.01 (− 0.05,0.07)	− 0.27 (− 0.34,− 0.19)	0.21 (0.15,0.27)
BMI	0.03 (0.007,0.06)	0.10 (0.07,0.13)	0.13 (0.10,0.15)	0.14 (0.11,0.16)	− 0.01 (− 0.04,0.02)	0.07 (0.04,0.10)	0.10 (0.07,0.13)	0.12 (0.09,0.15)	0.14 (0.12,0.16)
KTR				0.28 (0.25,0.30)	0.23 (0.20,0.26)	0.31 (0.28,0.33)	− 0.11 (− 0.14,− 0.08)	0.01 (− 0.02,0.04)	0.46 (0.44,0.49)
Neopterin	0.48 (0.45,0.51)	− 0.15 (− 0.18,− 0.12)	0.36 (0.33,0.38)	0.18 (0.14,0.21)	0.18 (0.15,0.22)	0.28 (0.25,0.31)	− 0.03 (− 0.06,0.006)	0.15 (0.11,0.18)	0.44 (0.41,0.46)
CRP	0.10 (0.07,0.12)	− 0.001 (− 0.03,0.03)	0.09 (0.07,0.12)	− 0.02 (− 0.05,0.005)	0.03 (− 0.0002,0.06)	0.06 (0.04,0.09)	− 0.03 (− 0.06,− 0.006)	0.11 (0.08,0.13)	0.14 (0.12,0.17)
Creatinine	0.28 (0.25,0.31)	0.04 (0.01,0.07)	0.33 (0.30,0.36)	0.47 (0.44,0.50)	0.16 (0.13,0.19)	0.30 (0.27,0.33)	0.31 (0.28,0.35)	0.10 (0.06,0.13)	0.35 (0.32,0.37)
Cotinine	− 0.05 (− 0.08,− 0.03)	0.01 (− 0.02,0.04)	− 0.05 (− 0.07,− 0.02)	− 0.02 (− 0.05,0.009)	− 0.13 (− 0.16,− 0.10)	− 0.07 (− 0.10,− 0.04)	− 0.04 (− 0.07,− 0.005)	− 0.04 (− 0.07,− 0.01)	− 0.16 (− 0.19,− 0.14)
Riboflavin	0.02 (− 0.01,0.05)	− 0.01 (− 0.04,0.02)	0.01 (− 0.01,0.04)	− 0.002 (− 0.03,0.03)	0.06 (0.03,0.09)	0.05 (0.02,0.08)	0.03 (0.004,0.06)	0.09 (0.06,0.12)	0.07 (0.05,0.10)
PLP	− 0.13 (− 0.16,− 0.11)	0.16 (0.13,0.19)	0.01 (− 0.01,0.04)	0.12 (0.09,0.15)	0.05 (0.02,0.08)	− 0.28 (− 0.31,− 0.25)	0.10 (0.07,0.13)	0.17 (0.14,0.20)	− 0.001 (− 0.03,0.02)

The numbes given are regression coefficients with 5,95% CI. The models were calculated by including cohorts and regions as random effects, and age, sex, BMI, CRP, KTR or neopterin (KTR and neopterin were included in separate models), creatinine, cotinine, riboflavin, PLP and sample storage time as fixed effects. All continuous variables were log-transformed and then centered on the mean and standardized by dividing by the standard deviation. For sex the regression coefficients represent the ratio of biomarker levels for women vs men.

AA: anthranilic acid; BMI: body mass index; CRP: C-reactive protein; HAA: 3-hydroxyanthranilic acid; HK: 3-hydroxykynurenine; KA: kynurenic acid; KTR: kynurenine/tryptophan ratio; PLP: pyridoxal 5′-phosphate; QA: quinolinic acid; XA: xanthurenic acid.

### Relative importance of KTR, neopterin and CRP as predictors of kynurenines

**Figure 3:**
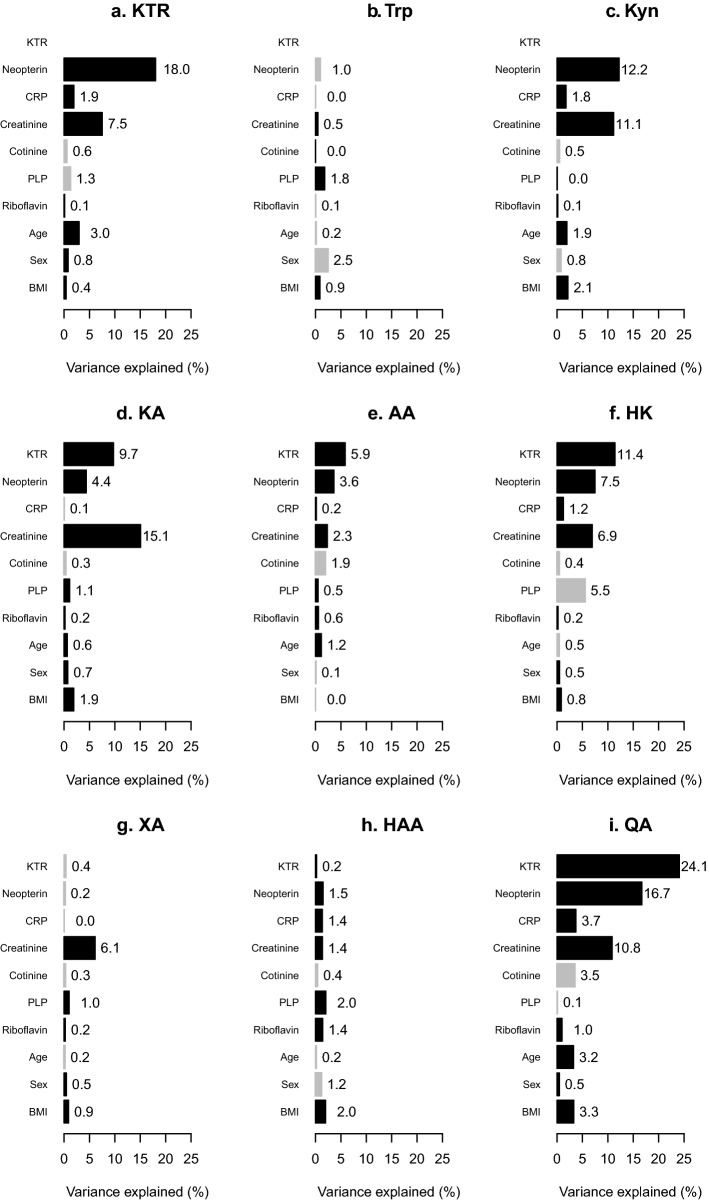
Relative importance of predictors of kynurenines. The percentage of variance of tryptophan and kynurenines explained by regression models using relaimpo. All continuous variables were log-transformed and then centered on the mean and standardized by dividing by the standard deviation. Black bars are used for predictors that showed positive regression coefficients in the mixed models (Table 2), those with negative coefficients are shown in grey. KTR and neopterin were included in separate models, and the models were adjusted for cohorts. AA: anthranilic acid; BMI: body mass index; CRP: C-reactive protein; HAA: 3-hydroxyanthranilic acid; HK: 3-hydroxykynurenine; KA: kynurenic acid; KTR: kynurenine/tryptophan ratio; PLP: pyridoxal 5′-phosphate; QA: quinolinic acid; XA: xanthurenic acid.

Using the relaimpo package, the highest relative importance as predictor was KTR explaining 24.1% of the variation of QA (Fig. 3, panel i). KTR explained 11.4% of the variation in HK and 9.7% of KA (Fig. 3, panels d and f). Neopterin explained 18.0% of the variation in KTR, 16.7% of QA, 12.2% of kynurenine and 7.5% of HK (Fig. 3, panels a, c, f and i). CRP predicted only a small amount of the variation in kynurenines and was largest at 3.7% for QA (Fig. 3, panel i). These same patterns of relative importance were obtained from calculations stratified by age (below and above the median age, Supplementary Figs. S1 and S2) and by smoking categories (never, former and current smokers, Supplementary Figs. S3–S5).

### Linearity of associations of KTR, neopterin and CRP with kynurenines

The associations of KTR with HK and QA in GAMs were nonlinear (Supplementary Fig. S6). Segmented regression showed that the steepest positive slopes were found for these KTR—metabolite relations at 0.1 and 0.3 SD above the geometric mean of KTR, respectively, with the steepest slope observed for KTR—QA, (Supplementary Table S4). In analysis below and above the median age, the breakpoints for KTR-HK and KTR-QA were found at higher values of KTR in the younger compared to the older age group (1.7 (95% CI 1.3 to 2.2) vs − 0.1 (95% CI − 0.6 to 0.5) and 1.4 (95% CI 1.1 to 1.7) vs − 0.8 (95% CI − 1.2 to − 0.4), respectively), Supplementary Table S5. For each relation, the slopes below the breakpoints were similar, while the slopes were steeper above the breakpoints in the younger group compared to the older group. No differences according to smoking categories were found, Supplementary Table S6.

GAM also showed nonlinear associations of neopterin with KTR, tryptophan and several kynurenines (Supplementary Fig. S7). From segmented regression, steeper positive relations were found for KTR, kynurenine, AA, HK and QA at neopterin higher than 0.2–0.9 SD above the geometric mean of neopterin (Supplementary Table S7). Steeper inverse slopes, though more gentle than the steepest positive slopes observed, were found for the associations of neopterin with tryptophan and XA, with breakpoints located at neopterin higher than 0.9 and 1.5 SD above the geometric mean of neopterin, respectively. In analysis by age groups the breakpoints were found at higher values of neopterin in the younger compared to the older age group for neopterin-KTR: 1.1 (95% CI 0.8 to 1.3) vs − 0.1 (95% CI − 0.4 to 0.3); neopterin-kynurenine: 0.9 (95% CI 0.6 to 1.2) vs − 0.4 (95% CI − 0.9 to 0.0) and neopterin-QA: 1.1 (95% CI 0.8 to 1.3) vs − 0.6 (95% CI − 0.9 to − 0.2), Supplementary Table S8. For each relation, the slopes below the breakpoints were similar, while the slopes were steeper above the breakpoints in the younger group compared to the older group. Few differences according to smoking categories were found, Supplementary Table S9.

CRP was nonlinearly related with kynurenine, KA and XA in the GAMs (Supplementary Fig. S8). Using segmented regression, positive relations of CRP with tryptophan and KA were found at CRP lower than 1.1 and 0.5 SD above the geometric mean, respectively, while the CRP–XA relation showed a slope that was not different from zero at CRP lower than 0.5 SD above the geometric mean of CRP, Supplementary Table S10. Above these break-points, these relations were inverse. The slopes involving CRP were generally more gentle than the slopes found for neopterin and KTR as predictors. The results for CRP as predictor showed that the breakpoint of the CRP-XA relation occured at higher CRP concentration in the younger compared to the older group (− 1.8 (95% − 2.3 to − 1.3) vs 0.9 (95% 0.4 to 1.4), respectively (Supplementary Table S11). The CRP-KA and CRP-XA relations showed significant breakpoints in the former smoking category only, Supplementary Table S12.

### Relations of creatinine with kynurenines

In the mixed models, 1SD higher creatinine was associated with 0.47 (95% CI 0.44 to 0.50) SD higher KA, and 0.28–0.35 SD higher KTR, kynurenine, HK, XA and QA (Table 2). Creatinine as predictor explained 15.1% of the variation of KA, followed by kynurenine (11.0%) and QA (10.9%), Fig. 3. The relations of creatinine with kynurenines were linear (Supplementary Fig. S9).

### Total variance explained by GAM and Relaimpo

The proportion of total variance explained by the GAM and Relaimpo methods were highest for the kynurenines that were most strongly related with KTR and neopterin in the regression models. The largest proportion of total variance explained was for QA at 49.6 to 56.2%, Supplementary Table S13. With models containing neopterin as predictor, 42.4% and 41.6% of the variance in kynurenine, while 42.7% and 41.1% of the variance in KTR were explained. For KA and HK the models explained 31.8–36.3% of their variance. For all outcomes except HAA, the models which included KTR explained a higher proportion of the total variance than did the models that included neopterin.

## Discussion

### Main findings

In this study we cross-sectionally investigated associations of KTR, neopterin and CRP with circulating concentrations of tryptophan metabolites of the kynurenine pathway in 5314 participants from 20 cohorts spanning the US, Nordic region, Asia and Australia. The results show that the association of inflammation with kynurenines, as quantified by the relations with KTR and neopterin, vary significantly for the investigated biomarkers. QA, followed by kynurenine and HK, were the metabolites most strongly associated with these IFN-γ induced markers of inflammation. CRP was only weakly related to QA, and essentially not related with the other kynurenines.

### Overall relations of neopterin and KTR with kynurenines

The positive correlations of neopterin with KTR^22,29,40–42^ and kynurenines^16,29,41–43^ observed in this study are in-line with existing reports. The results that KTR generally showed stronger associations with kynurenines than did neopterin, may result from KTR more closely reflecting the initiating step of the kynurenine pathway, while neopterin reflects IFN-γ activity^3^. In-line with this, the fraction of total variation of kynurenines explained by the GAM and Relaimpo models was larger in models that included KTR compared with models including neopterin.

### Metabolites along the tryptophan-NAD axis

In addition to resulting in increased kynurenine/tryptophan ratio (KTR)^12^ in serum/plasma, the increased formation of kynurenine from tryptophan was associated with increased concentrations of downstream metabolites of this pathway, including kynurenine^13^, HK^13^ and QA^12,13^. We found that QA was the metabolite which showed the strongest associations (in terms of correlations, regression coefficients, and importance of predictors) with KTR and neopterin. Studies have demonstrated that QA is produced by macrophages both without^44^, and with stimulation by IFN-γ^14,44–47^. IFN-γ combined with kynurenine caused a stronger increase in QA compared with the addition of only kynurenine in human macrophages^10^, suggesting that the effect of IFN-γ on QA levels is not caused only by increased IDO activity. IFN-γ is also involved in regulating enzymes in the kynurenine pathway other than IDO ^48^. Kynurenine mono-oxygenase^9–12^, kynureninase^9–11^ and HAAO^11^, i.e. all enzymes in the pathway leading to QA formation from kynurenine, were increased in macrophages stimulated with IFN-γ. One study reported finding no effect of IFN-γ on HAAO activity in macrophages^9^. The finding that picolinic acid was reduced while QA was increased by stimulating macrophages with IFN-γ, suggests that during immune stimulation, ACMSD expression may remain low causing a higher non-enzymatic conversion of ACMS to QA^45^. However, it has also been reported that QPRT was stimulated with IFN-γ^11,12^.

QA is the substrate for QPRT, which ultimately results in production of nicotinamide adenine dinucleotide (NAD)^49^. It has been suggested that a portion of kynurenine pathway metabolites may be directed toward replenishing cellular NAD levels in response to inflammation and infection, perhaps causing the high levels of QA in immune cells^15,50^. Administration of lipopolysaccharide to human monocyte-derived macrophages resulted in increased concentrations of kynurenine, HK, HAA and QA, as well as increases in IDO, kynurenine mono-oxygenase, kynureninase and HAAO, reduced QPRT, and increased de-novo NAD generation^8^. Additionally, exposure of macrophages to IFN-γ increased intracellular NAD concentrations, and this response was dependent on IDO activity and the presence of tryptophan in the cultural medium^51^. The results from the present and published work showing that kynurenine^29^, HK^29^ and QA were the metabolites that were most strongly associated with KTR and neopterin may support the idea that elevated QA reflects increased de novo synthesis of NAD during inflammation and immune activation.

Interestingly, GAMs and segmented regression showed that the relations of KTR and neopterin with metabolites along the tryptophan–NAD axis (kynurenine, HK and QA) were steeper at high compared with low/normal KTR and neopterin levels. These results might reflect IFN-γ being a stronger determinant of circulating levels of KTR, neopterin and kynurenines in inflammation, and accelerated formation of NAD when the degree of IFN-γ induced inflammation increases. Thus, most of the stronger associations with KTR and neopterin were for kynurenines located along the tryptophan-NAD axis, and these have also been found to be most strongly related with clinical outcomes such as cancer^17,23,24^ and cardiovascular disease^17,20^, though for all-cause mortality the results are not consistent^16,17^. In segmented regression models using KTR and neopterin as predictors, the differences in location of breakpoints, as well as steepness of the slopes above the breakpoints, in the younger vs older age groups, may be related to the increased level of inflammation^52^, KTR^29,38^, neopterin^29,38^ and kynurenines^29,38^ in aging. Further studies are needed to adress this issue.

### Metabolites along the side branches

KA and AA, which are formed directly from kynurenine, showed somewhat weaker positive associations with KTR and neopterin than did the metabolites along the tryptophan—NAD axis. XA showed no or very weak correlations with KTR and neopterin, and in the regression models XA was inversely related with KTR and neopterin. For these kynurenines experimental studies on the effects of stimulation of the immune response are scarce. It has been reported that plasma KA was reduced in mice after injection of pokeweed mitogen^13^, while human peripheral blood mononuclear cells showed no change in AA upon treatment with IFN-γ^12^. Results on the effect of stimulation of macrophages by IFN-γ on the expression of kynurenine aminotransferase, which catalyses the formation of KA from kynurenine and XA from HK, are conflicting^9,11^.

Epidemiologic studies have demonstrated that KA shows no^16,17^ while AA and XA show conflicting^16,17^ associations with all-cause mortality. A study of kynurenines as predictors of risk of lung cancer showed inconsistent results for KA, AA and XA depending on the models^24^; notably, none of the models included a marker of renal function, which is an important determinant of circulating concentrations of these metabolites^29^ (Table 2 and Fig. 3). In a large prospective study neither, KA, AA nor XA were associated with risk of acute coronary events^20^. Thus, compared with kynurenines along the tryptophan-NAD axis, the kynurenines on the side-branches appear to be less associated with inflammation and disease risk.

### Relations of CRP with kynurenines

This and previous epidemiologic works have demonstrated that CRP shows weak to moderate positive correlations with kynurenines^16,17^. In the regression models of kynurenines, which included either KTR or neopterin in combination with CRP, we found that CRP was only weakly or not associated with kynurenines. CRP is a non-specific marker of inflammation generated in response to interleukin-1β and interleukin-6^25^, and exposure to interleukin-6 has been found to increase IDO-1 expression and KTR^53^. The weak associations of CRP with kynurenines suggest that interleukin-6 is a weaker activator of IDO than is IFN-γ.

### Relations of creatinine with kynurenines

The strong positive relation of creatinine, which is often used as a marker of renal function, with kynurenines in this work, are in-line with the findings in cross-sectional studies of large Norwegian^29^ and Australian^16^ cohorts, and experimental renal failure in rats^54,55^, though in renal patients results are not consistent for all the included kynurenines^54,56^. In patients with chronic renal disease, of which inflammation is a central feature, plasma concentrations of kynurenine, KA and QA were found to be elevated when compared to healthy controls^57^, and to increase with increasing disease severity^58^.

The observed associations may be caused by the role of kidneys in excreting kynurenines and the activity of enzymes involved in this pathway in the kidneys^59^. Accordingly, when comparing rats with experimental renal failure with controls, higher activity of KAT and KMO, lower KYNU and HAAO, and no difference in IDO in kidneys were reported^55^. The same study found higher activities of liver TDO, KYNU and KMO, and lower KAT and HAAO^55^. In this and a previous^29^ study, KA was the kynurenine pathway metabolite that was most strongly related to renal function, and in the present work creatinine was the strongest predictor of KA. However, in a study that included patients with chronic renal failure, plasma KA was not different from controls^56^. After intraperitoneal injection of KA in mice, KA was most strongly accumulated in kidneys^60^. In a rat model of renal insufficiency, the increase in serum kynurenine and QA was proposed to be caused by reduced degradation and/or increased production of kynurenine and QA in combination with reduced renal excretion^54^. It has also been suggested that elevated serum kynurenine in human patients with renal failure might be caused by reduced renal clearance, perhaps combined with increased metabolism of tryptophan^61^. Though the kidneys are considered to be important in the metabolism of kynurenines^62^, many studies are limited by the fact that only a selection of kynurenines are included. Thus further studies are needed to increase our understanding of the role and importance of the kidneys in this pathway.

## Strengths and weaknesses

The main strength of this work include the large number of participants, combined with measurement of a large number of metabolites in the kynurenine pathway. In addition, we were able to adjust for several factors that are known to be related to circulating concentrations of kynurenines, including age^29,38^, sex^29,38^, BMI^29,38^, renal function^29^ and smoking^29,38^, as well as vitamins B2^63^ and B6^63^, which are cofactors for enzymes in this pathway. All the biomarkers were measured in the same laboratory, using mass-spectrometry based assays that included authentic isotope-labelled internal standards for each analyte, thus providing quantification with high precision compared with semiquantitative or untargeted methods^64^.

The inclusion of healthy subjects from 20 cohorts spanning four continents may suggest that the overall results are valid for healthy populations of the included age groups (middle-aged to old) in the included geographic regions. Further studies should be performed to confirm if this is the case and investigate how the investigated biomarkers are related in other populations.

The principal weaknesses are the inadequacy of a cross-sectional design to address causality, and that creatinine is an imperfect measure of renal function.

## Conclusions

In a study comprised of 5314 participants from four continents we cross-sectionally investigated the relations of markers of inflammation, KTR, neopterin and CRP, with circulating concentrations of metabolites in the kynurenine pathway. Regression models indicated that KTR and neopterin, which are increased mainly in response to stimulation by IFN-γ, were most strongly associated with QA, followed by kynurenine and HK. These metabolites are located along the tryptophan-NAD axis. Associations of CRP, which is formed in response to interleukin-1β and interleukin-6, with kynurenines were weak. The findings that KTR and neopterin were most strongly related with kynurenines along the tryptophan-NAD axis, and particularly with QA, add support to the idea of increased production of NAD from tryptophan during inflammation.

## Supplementary Information

Supplementary Information.

## Data Availability

Data from the Lung Cancer Cohort Consortium are not publicly available because approval for analysis must be obtained from each participating cohort. Researchers who are interested in analyzing the LC3 data are encouraged to contact Mattias Johansson at the International Agency for Research on Cancer (http://www.iarc.who.int).
