# Supplementary material for: A cross-sectional study of inflammatory markers as determinants of circulating kynurenines in the Lung Cancer Cohort Consortium
Source: Sci Rep. 2023 Jan 18;13:1011. doi: 10.1038/s41598-023-28135-9 (PMC9849351; doi:10.1038/s41598-023-28135-9)
Supplement: Supplementary file 1 — Supplementary Information. [file 41598_2023_28135_MOESM1_ESM.docx]

**A cross-sectional study of inflammatory markers as determinants of circulating kynurenines in the Lung Cancer Cohort Consortium**

Øivind Midttun^1^, Arve Ulvik^1^, Klaus Meyer^1^, Hana Zahed^2^, Graham G. Giles^3,4,5^, Jonas Manjer^6,7^, Malte Sandsveden^8^, Arnulf Langhammer^9,10^, Elin Pettersen Sørgjerd^9,11^, Annelie F Behndig^12^, Mikael Johansson^13^, Neal D. Freedman^14^, Wen-Yi Huang^14^, Chu Chen^15^, Ross Prentice^15^, Victoria L. Stevens^16^, Ying Wang^16^, Loïc Le Marchand^17^, Stephanie J. Weinstein^14^, Qiuyin Cai^18^, Alan A. Arslan^19,20,21^, Yu Chen ^20,21^, Xiao-Ou Shu^20^, Wei Zheng^20^, Jian-Min Yuan^22^, Woon-Puay Koh^23^, Kala Visvanathan^24^, Howard D. Sesso^25,26^, Xuehong Zhang^25,26^, J Michael Gaziano^27,28^, Anouar Fanidi^29^, and Hilary A. Robbins^2^, Paul Brennan^2^, Mattias Johansson^2^, Per M. Ueland^1^

^1^Bevital AS, Bergen, Norway,^2^Genomic Epidemiology Branch, International Agency For Research on Cancer, Lyon, France, ^3^Cancer Epidemiology Division, Cancer Council Victoria, Melbourne, Australia, ^4^Centre for Epidemiology and Biostatistics, School of Population and Global Health, The University of Melbourne, Melbourne, Australia, ^5^Precision Medicine, School of Clinical Sciences at Monash Health, Monash University, Melbourne, Australia, ^6^Department of Surgery, Skane University Hospital, Malmo, Sweden, ^7^Lund University, Malmo, Sweden, ^8^Department of Clinical Sciences Malmo, Lund University, Malmo, Sweden, ^9^Department of Public Health and Nursing, Hunt Research Centre, Norwegian University of Science and Technology, Levanger, Norway, ^10^Levanger Hospital , Nord-Trøndelag Hospital Trust, Levanger, Norway, ^11^Department of Endocrinology, St. Olavs Hospital, Trondheim University Hospital, Levanger, Norway, ^12^Department of Public Health and Clinical Medicine, Umea University, Umea, Sweden, ^13^Department of Radiation Sciences, Oncology, Umea University, Umea, Sweden, ^14^Metabolic Epidemiology Branch, Division of Cancer Epidemiology and Genetics, National Cancer Institute, Bethesda, Maryland, USA, ^15^Public Health Sciences Division, Fred Hutchinson Cancer Center, Seattle, USA, ^16^American Cancer Society, Atlanta, USA, ^17^University of Hawai'i Cancer Center, University of Hawaiʻi at Mānoa, Honolulu, USA, ^18^Vanderbilt University Medical Center, Nashville, USA, ^19^Department of Obstetrics and Gynecology, NYU Langone Health, New York, NY, USA, ^20^Department of Population Health, NYU Langone Health, New York, NY, USA, ^21^Perlmutter Comprehensive Cancer Center, NYU Langone Health, New York, NY, USA, ^22^University of Pittsburgh and UPMC Hillman Cancer Center, Pittsburgh, USA, ^23^Healthy Longevity Translational Research Programme, Yong Loo Lin School of Medicine, National University of Singapore, Singapore, ^24^Johns Hopkins Institute for Clinical and Translational Research, Baltimore, USA, ^25^Brigham and Women's Hospital, Harvard Medical School, Boston, USA, ^26^Harvard T.H. Chan School of Public Health, Boston, USA, ^27^Brigham and Women's Hospital, Boston, USA, ^28^VA Boston Healthcare System, Boston, MA, USA, ^29^Université Claude Bernard Lyon 1, Lyon, France

Supplementary Table S1. Spearman correlations adjusted for cohort, age, sex, BMI, creatinine, cotinine and sample storage time^1^

|  | Neo | CRP | Trp | Kyn | KA | AA | HK | XA | HAA | QA | Rf | PLP |
| --- | --- | --- | --- | --- | --- | --- | --- | --- | --- | --- | --- | --- |
| KTR | 0.38 | 0.16 | -0.50 | 0.52 | 0.24 | 0.23 | 0.32 | -0.09 | 0.00* | 0.46 | -0.04* | -0.19 |
| Neo |  | 0.13 | -0.11 | 0.31 | 0.13 | 0.17 | 0.25 | -0.01* | 0.10 | 0.38 | -0.01* | -0.11 |
| CRP |  |  | -0.03* | 0.15 | 0.00* | 0.06 | 0.14 | -0.03* | 0.12 | 0.23 | 0.02* | -0.15 |
| Trp |  |  |  | 0.39 | 0.12 | 0.07 | 0.10 | 0.34 | 0.40 | 0.07 | 0.05 | 0.18 |
| Kyn |  |  |  |  | 0.38 | 0.32 | 0.45 | 0.24 | 0.40 | 0.59 | 0.01* | -0.03* |
| KA |  |  |  |  |  | 0.29 | 0.35 | 0.58 | 0.41 | 0.28 | 0.03* | 0.11 |
| AA |  |  |  |  |  |  | 0.12 | 0.12 | 0.20 | 0.31 | 0.07 | 0.06 |
| HK |  |  |  |  |  |  |  | 0.41 | 0.43 | 0.43 | -0.06 | -0.29 |
| XA |  |  |  |  |  |  |  |  | 0.53 | 0.20 | 0.06 | 0.12 |
| HAA |  |  |  |  |  |  |  |  |  | 0.40 | 0.14 | 0.14 |
| QA |  |  |  |  |  |  |  |  |  |  | 0.08 | -0.02* |
| Rf |  |  |  |  |  |  |  |  |  |  |  | 0.34 |

^1^All correlations without an asterix are significant with p<0.001. AA: anthranilic acid; BMI: body mass index; CRP: C-reactive protein; HAA: 3-hydroxyanthranilic acid; HK: 3-hydroxykynurenine; KA: kynurenic acid; KTR: kynurenine/tryptophan ratio; Kyn: kynurenine; Neo: neopterin; PLP: pyridoxal 5’-phosphate; Rf: riboflavin; Trp: tryptophan; QA: quinolinic acid; XA: xanthurenic acid.

Supplementary Table S2. Predictors of kynureniners and KTR by mixed models by age groups below and above median age (62 years)^1^

|  | Age group | KTR | Tryptophan | Kynurenine | KA | AA | HK | XA | HAA | QA |
| --- | --- | --- | --- | --- | --- | --- | --- | --- | --- | --- |
| Age | <median | 0.07 (0.03,0.10) | -0.003 (-0.04,0.04) | 0.06 (0.03,0.10) | 0.04 (0.002,0.08) | 0.05 (0.007,0.09) | 0.002 (-0.03,0.04) | 0.01 (-0.03,0.05) | -0.07 (-0.11,-0.03) | 0.05 (0.01,0.08) |
|  | >median | 0.09 (0.05,0.13) | -0.07 (-0.11,-0.03) | 0.03 (-0.01,0.06) | 0.02 (-0.02,0.06) | 0.02 (-0.03,0.07) | 0.02 (-0.02,0.06) | -0.07 (-0.11,-0.02) | -0.05 (-0.10,-0.01) | 0.09 (0.06,0.13) |
| Sex | <median | 0.36 (0.25,0.47) | -0.47 (-0.59,-0.36) | -0.03 (-0.11,0.05) | 0.05 (-0.04,0.15) | 0.02 (-0.06,0.09) | 0.27 (0.15,0.38) | -0.04 (-0.12,0.034) | -0.32 (-0.44,-0.20) | 0.15 (0.04,0.25) |
|  | >median | 0.34 (0.24,0.43) | -0.39 (-0.49,-0.28) | 0.01 (-0.06,0.08) | 0.11 (0.02,0.20) | -0.02 (-0.13,0.07) | 0.28 (0.19,0.38) | 0.08 (-0.009,0.17) | -0.18 (-0.28,-0.08) | 0.24 (0.16,0.33) |
| BMI | <median | 0.04 (0.0002,0.08) | 0.11 (0.07,0.15) | 0.15 (0.11,0.18) | 0.15 (0.11,0.19) | 0.009 (-0.04,0.05) | 0.09 (0.05,0.12) | 0.11 (0.07,0.16) | 0.14 (0.10,0.18) | 0.19 (0.15,0.22) |
|  | >median | 0.04 (0.004,0.08) | 0.08 (0.04,0.13) | 0.12 (0.08,0.16) | 0.14 (0.10,0.18) | -0.02 (-0.07,0.02) | 0.05 (0.01,0.09) | 0.08 (0.04,0.12) | 0.10 (0.06,0.15) | 0.12 (0.08,0.15) |
| KTR | <median |  |  |  | 0.27 (0.23,0.31) | 0.23 (0.19,0.27) | 0.29 (0.25,0.33) | -0.09 (-0.13,-0.04) | 0.03 (-0.01,0.07) | 0.45 (0.42,0.49) |
|  | >median |  |  |  | 0.27 (0.23,0.30) | 0.22 (0.17,0.26) | 0.32 (0.28,0.36) | -0.11 (-0.16,-0.07) | -0.008 (-0.05,0.03) | 0.47 (0.44,0.51) |
| Neopterin | <median | 0.48 (0.44,0.52) | -0.14 (-0.19,-0.10) | 0.35 (0.31,0.39) | 0.20 (0.15,0.24) | 0.14 (0.09,0.19) | 0.30 (0.26,0.35) | 0.02 (-0.03,0.07) | 0.18 (0.14,0.23) | 0.44 (0.39,0.48) |
|  | >median | 0.48 (0.44,0.52) | -0.15 (-0.20,-0.11) | 0.36 (0.32,0.40) | 0.15 (0.11,0.20) | 0.22 (0.17,0.27) | 0.26 (0.21,0.30) | -0.07 (-0.12,-0.02) | 0.10 (0.06,0.15) | 0.43 (0.39,0.47) |
| CRP | <median | 0.10 (0.07,0.14) | -0.002 (-0.04,0.04) | 0.10 (0.07,0.14) | -0.03 (-0.07,0.006) | 0.03 (-0.01,0.07) | 0.05 (0.02,0.09) | -0.04 (-0.08,0.001) | 0.09 (0.05,0.13) | 0.13 (0.10,0.17) |
|  | >median | 0.09 (0.06,0.12) | -0.01 (-0.05,0.03) | 0.08 (0.05,0.12) | -0.02 (-0.06,0.01) | 0.02 (-0.02,0.07) | 0.06 (0.03,0.10) | -0.04 (-0.08,0.001) | 0.11 (0.07,0.15) | 0.16 (0.13,0.19) |
| Creatinine | <median | 0.27 (0.23,0.31) | 0.04 (-0.007,0.09) | 0.32 (0.28,0.36) | 0.41 (0.37,0.46) | 0.16 (0.12,0.21) | 0.25 (0.20,0.29) | 0.26 (0.22,0.31) | 0.08 (0.02,0.11) | 0.34 (0.30,0.38) |
|  | >median | 0.29 (0.25,0.33) | 0.05 (0.003,0.10) | 0.35 (0.31,0.39) | 0.51 (0.47,0.55) | 0.17 (0.12,0.21) | 0.34 (0.30,0.39) | 0.37 (0.32,0.41) | 0.13 (0.09,0.18) | 0.37 (0.33,0.41) |
| Cotinine | <median | -0.07 (-0.11,-0.04) | 0.01 (-0.03,0.06) | -0.06 (-0.10,-0.02) | -0.03 (-0.07,0.01) | -0.14 (-0.18,-0.09) | -0.08 (-0.12,-0.04) | -0.06 (-0.11,-0.02) | -0.02 (-0.06,0.03) | -0.18 (-0.22,-0.15) |
|  | >median | -0.010 (-0.05,0.03) | -0.04 (-0.08,-0.01) | -0.02 (-0.06,0.02) | -0.11 (-0.16,-0.07) | -0.06 (-0.10,-0.02) | -0.03 (-0.08,0.008) | -0.06 (-0.11,-0.02) | -0.14 (-0.17,-0.11) | -0.04 (-0.07,0.001) |
| Riboflavin | <median | -0.007 (-0.05,0.03) | 0.01 (-0.03,0.06) | 0.005 (-0.03,0.04) | -0.02 (-0.06,0.03) | 0.06 (0.01,0.10) | 0.06 (0.02,0.10) | 0.01 (-0.03,0.058) | 0.09 (0.05,0.13) | 0.08 (0.05,0.12) |
|  | >median | -0.02 (-0.06,0.03) | 0.02 (-0.02,0.05) | 0.01 (-0.03,0.05) | 0.05 (0.007,0.09) | 0.03 (-0.005,0.07) | 0.05 (0.005,0.09) | 0.08 (0.04,0.12) | 0.06 (0.03,0.09) | 0.03 (-0.006,0.07) |
| PLP | <median | -0.13 (-0.17,-0.09) | 0.16 (0.11,0.20) | 0.02 (-0.02,0.06) | 0.15 (0.11,0.19) | 0.04 (-0.01,0.09) | -0.27 (-0.31,-0.23) | 0.12 (0.08,0.17) | 0.16 (0.11,0.20) | 0.002 (-0.04,0.04) |
|  | >median | -0.13 (-0.16,-0.09) | 0.16 (0.11,0.20) | 0.01 (-0.03,0.05) | 0.09 (0.05,0.13) | 0.06 (0.01,0.10) | -0.29 (-0.33,-0.24) | 0.09 (0.04,0.13) | 0.18 (0.14,0.23) | 0.002 (-0.03,0.04) |

^1^ The numbes given are regression coefficients with 5,95% CI. The models were calculated by including cohorts and regions as random effects, and age, sex, BMI, CRP, KTR or neopterin (KTR and neopterin were included in separate models), creatinine, cotinine, riboflavin, PLP and sample storage time as fixed effects. All continuous variables were log-transformed and then centered on the mean and standardized by dividing by the standard deviation. For sex the regression coefficients represent the ratio of biomarker levels for women vs men. AA: anthranilic acid; BMI: body mass index; CRP: C-reactive protein; HAA: 3-hydroxyanthranilic acid; HK: 3-hydroxykynurenine; KA: kynurenic acid; KTR: kynurenine/tryptophan ratio; PLP: pyridoxal 5’-phosphate; QA: quinolinic acid; XA: xanthurenic acid.

Supplementary Table S3. Predictors of kynureniners and KTR by mixed models by smoking categories^1^

|  | Smoking category | KTR | Tryptophan | Kynurenine | KA | AA | HK | XA | HAA | QA |
| --- | --- | --- | --- | --- | --- | --- | --- | --- | --- | --- |
| Age | Never smokers | 0.08 (0.03,0.14) | 0.05 (-0.01,0.11) | 0.13 (0.08,0.19) | 0.08 (0.02,0.14) | 0.08 (0.02,0.15) | -0.01 (-0.07,0.05) | -0.02 (-0.08,0.05) | -0.10 (-0.17,-0.04) | 0.09 (0.04,0.14) |
|  | Former smokers | 0.15 (0.10,0.20) | -0.08 (-0.14,-0.02) | 0.08 (0.03,0.14) | 0.03 (-0.03,0.08) | 0.05 (-0.009,0.11) | -0.001 (-0.05,0.05) | -0.12 (-0.18,-0.06) | -0.08 (-0.14,-0.02) | 0.14 (0.09,0.19) |
|  | Current smokers | 0.11 (0.07,0.14) | -0.05 (-0.09,-0.005) | 0.07 (0.03,0.11) | 0.05 (0.01,0.09) | 0.10 (0.06,0.14) | 0.01 (-0.02,0.05) | -0.02 (-0.06,0.02) | -0.09 (-0.13,-0.04) | 0.09 (0.05,0.12) |
| Sex | Never smokers | 0.38 (0.21,0.54) | -0.31 (-0.49,-0.12) | 0.06 (-0.03,0.16) | 0.05 (-0.09,0.21) | 0.06 (-0.07,0.17) | 0.43 (0.25,0.60) | 0.05 (-0.05,0.16) | -0.14 (-0.30,0.012) | 0.16 (-0.005,0.32) |
|  | Former smokers | 0.34 (0.22,0.45) | -0.42 (-0.57,-0.28) | -0.01 (-0.10,0.07) | 0.02 (-0.06,0.10) | 0.02 (-0.07,0.12) | 0.18 (0.07,0.29) | 0.003 (-0.08,0.09) | -0.25 (-0.38,-0.12) | 0.17 (0.06,0.28) |
|  | Current smokers | 0.41 (0.31,0.51) | -0.43 (-0.55,-0.31) | 0.01 (-0.06,0.09) | 0.14 (0.03,0.24) | -0.05 (-0.12,0.04) | 0.33 (0.22,0.44) | -0.007 (-0.08,0.07) | -0.24 (-0.36,-0.13) | 0.24 (0.14,0.34) |
| BMI | Never smokers | 0.04 (-0.02,0.09) | 0.08 (0.02,0.15) | 0.12 (0.06,0.17) | 0.10 (0.04,0.17) | -0.01 (-0.08,0.05) | 0.03 (-0.04,0.09) | 0.02 (-0.04,0.09) | 0.09 (0.02,0.16) | 0.12 (0.07,0.18) |
|  | Former smokers | 0.06 (0.016,0.10) | 0.04 (-0.01,0.10) | 0.10 (0.06,0.15) | 0.16 (0.11,0.20) | 0.009 (-0.04,0.07) | 0.06 (0.01,0.10) | 0.05 (-0.002,0.10) | 0.11 (0.06,0.16) | 0.12 (0.08,0.16) |
|  | Current smokers | 0.004 (-0.03,0.04) | 0.14 (0.10,0.18) | 0.13 (0.10,0.17) | 0.12 (0.09,0.16) | -0.02 (-0.06,0.02) | 0.08 (0.04,0.12) | 0.13 (0.09,0.18) | 0.13 (0.09,0.17) | 0.15 (0.12,0.19) |
| KTR | Never smokers |  |  |  | 0.28 (0.22,0.34) | 0.25 (0.18,0.32) | 0.30 (0.24,0.37) | -0.09 (-0.15,-0.02) | -0.02 (-0.08,0.05) | 0.47 (0.42,0.52) |
|  | Former smokers |  |  |  | 0.28 (0.24,0.33) | 0.24 (0.19,0.30) | 0.36 (0.31,0.41) | -0.16 (-0.21,-0.10) | 0.02 (-0.03,0.07) | 0.51 (0.46,0.55) |
|  | Current smokers |  |  |  | 0.27 (0.23,0.31) | 0.21 (0.16,0.25) | 0.26 (0.22,0.30) | -0.08 (-0.12,-0.03) | 0.005 (-0.04,0.05) | 0.44 (0.41,0.47) |
| Neopterin | Never smokers | 0.42 (0.35,0.48) | -0.14 (-0.21,-0.07) | 0.31 (0.25,0.37) | 0.17 (0.10,0.24) | 0.20 (0.13,0.26) | 0.24 (0.17,0.31) | -0.02 (-0.08,0.05) | 0.13 (0.06,0.20) | 0.40 (0.34,0.46) |
|  | Former smokers | 0.53 (0.48,0.59) | -0.17 (-0.23,-0.10) | 0.40 (0.34,0.45) | 0.15 (0.10,0.21) | 0.23 (0.16,0.29) | 0.30 (0.25,0.36) | -0.09 (-0.16,-0.03) | 0.10 (0.04,0.17) | 0.47 (0.42,0.52) |
|  | Current smokers | 0.47 (0.42,0.51) | -0.14 (-0.19,-0.10) | 0.35 (0.31,0.39) | 0.18 (0.14,0.23) | 0.15 (0.10,0.20) | 0.28 (0.23,0.32) | 0.001 (-0.05,0.05) | 0.17 (0.13,0.22) | 0.44 (0.40,0.48) |
| CRP | Never smokers | 0.09 (0.04,0.14) | -0.03 (-0.10,0.02) | 0.07 (0.02,0.12) | -0.02 (-0.07,0.04) | 0.04 (-0.02,0.10) | -0.007 (-0.06,0.05) | -0.04 (-0.10,0.02) | 0.12 (0.06,0.18) | 0.14 (0.09,0.19) |
|  | Former smokers | 0.11 (0.07,0.16) | -0.004 (-0.06,0.05) | 0.11 (0.07,0.16) | 0.01 (-0.04,0.06) | 0.02 (-0.03,0.08) | 0.12 (0.08,0.17) | -0.001 (-0.05,0.05) | 0.14 (0.09,0.19) | 0.18 (0.14,0.22) |
|  | Current smokers | 0.09 (0.06,0.12) | 0.01 (-0.03,0.05) | 0.10 (0.07,0.14) | -0.04 (-0.07,-0.001) | 0.03 (-0.02,0.07) | 0.06 (0.02,0.09) | -0.05 (-0.09,-0.01) | 0.08 (0.04,0.12) | 0.14 (0.11,0.17) |
| Creatinine | Never smokers | 0.33 (0.26,0.39) | 0.03 (-0.04,0.10) | 0.37 (0.32,0.43) | 0.44 (0.37,0.50) | 0.20 (0.13,0.27) | 0.40 (0.33,0.47) | 0.31 (0.24,0.37) | 0.13 (0.06,0.2) | 0.34 (0.27,0.40) |
|  | Former smokers | 0.23 (0.18,0.28) | 0.05 (-0.005,0.11) | 0.30 (0.25,0.35) | 0.48 (0.43,0.53) | 0.16 (0.11,0.22) | 0.28 (0.23,0.33) | 0.34 (0.28,0.39) | 0.11 (0.06,0.17) | 0.30 (0.25,0.34) |
|  | Current smokers | 0.28 (0.24,0.32) | 0.03 (-0.02,0.07) | 0.31 (0.27,0.35) | 0.43 (0.39,0.47) | 0.14 (0.10,0.18) | 0.25 (0.20,0.29) | 0.27 (0.23,0.31) | 0.07 (0.03,0.11) | 0.36 (0.33,0.40) |
| Cotinine | Never smokers | -0.03 (-0.08,0.02) | 0.04 (-0.02,0.10) | 0.001 (-0.05,0.05) | 0.01 (-0.04,0.07) | 0.01 (-0.05,0.07) | -0.06 (-0.12,-0.005) | -0.03 (-0.09,0.03) | -0.02 (-0.08,0.04) | -0.03 (-0.08,0.02) |
|  | Former smokers | -0.003 (-0.05,0.04) | -0.009 (-0.06,0.04) | -0.010 (-0.05,0.03) | -0.006 (-0.05,0.04) | -0.03 (-0.08,0.03) | -0.03 (-0.07,0.02) | -0.03 (-0.08,0.02) | -0.01 (-0.06,0.04) | -0.06 (-0.10,-0.02) |
|  | Current smokers | -0.06 (-0.10,-0.03) | -0.001 (-0.04,0.04) | -0.07 (-0.10,-0.04) | -0.05 (-0.08,-0.01) | -0.09 (-0.13,-0.05) | -0.05 (-0.08,-0.01) | -0.06 (-0.10,-0.02) | -0.009 (-0.05,0.03) | -0.11 (-0.14,-0.08) |
| Riboflavin | Never smokers | 0.05 (-0.005,0.10) | -0.04 (-0.10,0.02) | 0.008 (-0.04,0.06) | -0.02 (-0.08,0.03) | 0.05 (-0.01,0.11) | 0.07 (0.02,0.13) | 0.03 (-0.03,0.09) | 0.10 (0.03,0.16) | 0.08 (0.03,0.12) |
|  | Former smokers | -0.007 (-0.06,0.04) | 0.03 (-0.03,0.09) | 0.02 (-0.03,0.07) | 0.005 (-0.05,0.06) | 0.09 (0.03,0.15) | 0.05 (-0.001,0.10) | 0.07 (0.009,0.12) | 0.13 (0.08,0.19) | 0.06 (0.01,0.11) |
|  | Current smokers | 0.02 (-0.02,0.05) | -0.007 (-0.05,0.03) | 0.01 (-0.02,0.05) | 0.006 (-0.03,0.04) | 0.05 (0.009,0.09) | 0.04 (0.006,0.08) | 0.03 (-0.01,0.07) | 0.07 (0.03,0.11) | 0.08 (0.05,0.12) |
| PLP | Never smokers | -0.13 (-0.18,-0.07) | 0.12 (0.06,0.19) | -0.02 (-0.08,0.03) | 0.17 (0.11,0.23) | 0.04 (-0.03,0.11) | -0.29 (-0.35,-0.23) | 0.09 (0.03,0.15) | 0.19 (0.13,0.26) | 0.02 (-0.03,0.07) |
|  | Former smokers | -0.11 (-0.15,-0.06) | 0.14 (0.08,0.20) | 0.02 (-0.03,0.071) | 0.11 (0.06,0.16) | 0.04 (-0.02,0.10) | -0.28 (-0.33,-0.22) | 0.07 (0.01,0.13) | 0.16 (0.11,0.22) | -0.008 (-0.05,0.04) |
|  | Current smokers | -0.13 (-0.17,-0.10) | 0.17 (0.13,0.21) | 0.02 (-0.02,0.06) | 0.09 (0.06,0.13) | 0.04 (-0.001,0.09) | -0.25 (-0.29,-0.22) | 0.10 (0.06,0.14) | 0.15 (0.11,0.19) | -0.007 (-0.04,0.03) |

^1^ The numbes given are regression coefficients with 5,95% CI. The models were calculated by including cohorts and regions as random effects, and age, sex, BMI, CRP, KTR or neopterin (KTR and neopterin were included in separate models), creatinine, cotinine, riboflavin, PLP and sample storage time as fixed effects. All continuous variables were log-transformed and then centered on the mean and standardized by dividing by the standard deviation. For sex the regression coefficients represent the ratio of biomarker levels for women vs men. AA: anthranilic acid; BMI: body mass index; CRP: C-reactive protein; HAA: 3-hydroxyanthranilic acid; HK: 3-hydroxykynurenine; KA: kynurenic acid; KTR: kynurenine/tryptophan ratio; PLP: pyridoxal 5’-phosphate; QA: quinolinic acid; XA: xanthurenic acid.

Supplemental Supplementary Figure S1. Relative importance of predictors of kynurenines in participants below median age (62 years). The percentage of variance of tryptophan and kynurenines explained by regression models using relaimpo. All continuous variables were log-transformed and then centered on the mean and standardized by dividing by the standard deviation. Black bars are used for predictors that showed positive regression coefficients in the mixed models (Table 2), those with negative coefficients are shown in grey. KTR and neopterin were included in separate models, and the models were adjusted for cohorts. AA: anthranilic acid; BMI: body mass index; CRP: C-reactive protein; HAA: 3-hydroxyanthranilic acid; HK: 3-hydroxykynurenine; KA: kynurenic acid; KTR: kynurenine/tryptophan ratio; Kyn: kynurenine; PLP: pyridoxal 5’-phosphate; Trp: tryptophan; QA: quinolinic acid; XA: xanthurenic acid.

Supplementary Figure S2. Relative importance of predictors of kynurenines in participants above median age (62 years). The percentage of variance of tryptophan and kynurenines explained by regression models using relaimpo. All continuous variables were log-transformed and then centered on the mean and standardized by dividing by the standard deviation. Black bars are used for predictors that showed positive regression coefficients in the mixed models (Table 2), those with negative coefficients are shown in grey. KTR and neopterin were included in separate models, and the models were adjusted for cohorts. AA: anthranilic acid; BMI: body mass index; CRP: C-reactive protein; HAA: 3-hydroxyanthranilic acid; HK: 3-hydroxykynurenine; KA: kynurenic acid; KTR: kynurenine/tryptophan ratio; Kyn: kynurenine; PLP: pyridoxal 5’-phosphate; Trp: tryptophan; QA: quinolinic acid; XA: xanthurenic acid.

Supplementary Supplementary Figure S3. Relative importance of predictors of kynurenines in never smoker participants. The percentage of variance of tryptophan and kynurenines explained by regression models using relaimpo. All continuous variables were log-transformed and then centered on the mean and standardized by dividing by the standard deviation. Black bars are used for predictors that showed positive regression coefficients in the mixed models (Table 2), those with negative coefficients are shown in grey. KTR and neopterin were included in separate models, and the models were adjusted for cohorts. AA: anthranilic acid; BMI: body mass index; CRP: C-reactive protein; HAA: 3-hydroxyanthranilic acid; HK: 3-hydroxykynurenine; KA: kynurenic acid; KTR: kynurenine/tryptophan ratio; Kyn: kynurenine; PLP: pyridoxal 5’-phosphate; Trp: tryptophan; QA: quinolinic acid; XA: xanthurenic acid.

Supplementary Figure S4. Relative importance of predictors of kynurenines in former smoker participants. The percentage of variance of tryptophan and kynurenines explained by regression models using relaimpo. All continuous variables were log-transformed and then centered on the mean and standardized by dividing by the standard deviation. Black bars are used for predictors that showed positive regression coefficients in the mixed models (Table 2), those with negative coefficients are shown in grey. KTR and neopterin were included in separate models, and the models were adjusted for cohorts. AA: anthranilic acid; BMI: body mass index; CRP: C-reactive protein; HAA: 3-hydroxyanthranilic acid; HK: 3-hydroxykynurenine; KA: kynurenic acid; KTR: kynurenine/tryptophan ratio; Kyn: kynurenine; PLP: pyridoxal 5’-phosphate; Trp: tryptophan; QA: quinolinic acid; XA: xanthurenic acid.

Supplementary Figure S5. Relative importance of predictors of kynurenines in current smoker participants. The percentage of variance of tryptophan and kynurenines explained by regression models using relaimpo. All continuous variables were log-transformed and then centered on the mean and standardized by dividing by the standard deviation. Black bars are used for predictors that showed positive regression coefficients in the mixed models (Table 2), those with negative coefficients are shown in grey. KTR and neopterin were included in separate models, and the models were adjusted for cohorts. AA: anthranilic acid; BMI: body mass index; CRP: C-reactive protein; HAA: 3-hydroxyanthranilic acid; HK: 3-hydroxykynurenine; KA: kynurenic acid; KTR: kynurenine/tryptophan ratio; Kyn: kynurenine; PLP: pyridoxal 5’-phosphate; Trp: tryptophan; QA: quinolinic acid; XA: xanthurenic acid.

Supplementary Figure S6. KTR as predictor of tryptophan and kynurenines by Generalized Additive Models (GAM).The models contained cohort, age, sex, BMI, KTR, CRP, creatinine, cotinine, PLP, riboflavin and sample storage time as predictors. All continuous variables were log-transformed and then centered on the mean and standardized by dividing by the standard deviation. AA: anthranilic acid; CRP: C-reactive protein; HAA: 3-hydroxyanthranilic acid; HK: 3-hydroxykynurenine; KA: kynurenic acid; KTR: kynurenine/tryptophan ratio; PLP: pyridoxal 5’-phosphate; QA: quinolinic acid; XA: xanthurenic acid.

Supplementary Table S4. Relations of KTR with kynurenines by Davies test and segmented regression^1^

|  | Davies test | |  | Segmented regression^2^ | | |
| --- | --- | --- | --- | --- | --- | --- |
|  | Breakpoint^3^ | p-value |  | Lower segment | Breakpoint^3^ | Upper segment |
|  |  |  |  | Slope (95% CI) |  | Slope (95% CI) |
| KA | 0.5 | 0.17 |  |  |  |  |
| AA | 4.8 | 0.33 |  |  |  |  |
| HK | 0.5 | 1.9E-04 |  | 0.20 (0.15, 0.26) | 0.1 (-0.4, 0.5) | 0.37 (0.32, 0.42) |
| XA | 3.1 | 0.73 |  |  |  |  |
| HAA | 0.5 | 0.07 |  |  |  |  |
| QA | 0.5 | 9.3E-11 |  | 0.36 (0.32, 0.4) | 0.3 (0.0, 0.6) | 0.58 (0.53, 0.63) |

^1^Adjusted for study center, age, sex, BMI, cotinine, creatinine, CRP, PLP, riboflavin and sample storage time. All continuous variables were log-transformed and then centered on the mean and standardized by dividing by the standard deviation. AA: anthranilic acid; CRP: C-reactive protein; HAA: 3-hydroxyanthranilic acid; HK: 3-hydroxykynurenine; KA: kynurenic acid; PLP: pyridoxal 5’-phosphate; QA: quinolinic acid; XA: xanthurenic acid.

^2^Results from segmented regression are shown only for biomarkers where Davies test gave a significant breakpoint (p-value <0.05).

^3^Breakpoints are given in SD units relative to the geometric mean of KTR.

Supplementary Table S5. Relations of KTR with kynurenines by Davies test and segmented regression by age group^1^

|  |  | Davies test | |  | Segmented regression^2^ | | |
| --- | --- | --- | --- | --- | --- | --- | --- |
|  | Age | Breakpoint^3^ | p-value |  | Lower segment | Breakpoint^3^ | Upper segment |
|  | group |  |  |  | Slope 1 (95% CI) |  | Slope 2 (95% CI) |
| KA | <median | 3.3 | 0.14 |  |  |  |  |
| KA | >median | -0.3 | 0.32 |  |  |  |  |
| AA | <median | 2.4 | 0.01 |  | 0.20 (0.15, 0.24) | 2.1 (1.4, 2.8) | 0.71 (0.36, 1.05) |
| AA | >median | 4.3 | 0.45 |  |  |  |  |
| HK | <median | 1.5 | 3.9E-04 |  | 0.21 (0.17, 0.26) | 1.7 (1.3, 2.2) | 0.68 (0.46, 0.91) |
| HK | >median | -0.3 | 0.04 |  | 0.19 (0.10, 0.29) | -0.1 (-0.6, 0.5) | 0.39 (0.33, 0.46) |
| XA | <median | 3.3 | 0.58 |  |  |  |  |
| XA | >median | 4.3 | 0.93 |  |  |  |  |
| HAA | <median | -2.1 | 0.17 |  |  |  |  |
| HAA | >median | 1.2 | 0.65 |  |  |  |  |
| QA | <median | 1.5 | 1.1E-09 |  | 0.36 (0.32, 0.40) | 1.4 (1.1, 1.7) | 0.90 (0.74, 1.07) |
| QA | >median | -1.1 | 0.01 |  | 0.25 (0.09, 0.41) | -0.8 (-1.2, -0.4) | 0.52 (0.48, 0.56) |

^1^Adjusted for study center, age, sex, BMI, cotinine, creatinine, CRP, PLP, riboflavin and sample storage time. All continuous variables were log-transformed and then centered on the mean and standardized by dividing by the standard deviation. AA: anthranilic acid; CRP: C-reactive protein; HAA: 3-hydroxyanthranilic acid; HK: 3-hydroxykynurenine; KA: kynurenic acid; KTR: kynurenine/tryptophan ratio; PLP: pyridoxal 5’-phosphate; QA: quinolinic acid; XA: xanthurenic acid.

^2^Results from segmented regression are shown only for biomarkers where Davies test gave a significant breakpoint (p-value <0.05).

^3^Breakpoints are given in SD units relative to the geometric mean of KTR.

Supplementary Table S6. Relations of KTR with kynurenines by Davies test and segmented regression by smoking category^1^

|  |  | Davies test | |  | Segmented regression^2^ | | |
| --- | --- | --- | --- | --- | --- | --- | --- |
|  | Smoking | Breakpoint^3^ | p-value |  | Lower segment | Breakpoint^3^ | Upper segment |
|  | category |  |  |  | Slope 1 (95% CI) |  | Slope 2 (95% CI) |
| KA | Never | -2.6 | 0.47 |  |  |  |  |
| KA | Former | -1.2 | 0.34 |  |  |  |  |
| KA | Current | 0.6 | 0.12 |  |  |  |  |
| AA | Never | -2.6 | 0.62 |  |  |  |  |
| AA | Former | -2.6 | 0.65 |  |  |  |  |
| AA | Current | 3.9 | 0.12 |  |  |  |  |
| HK | Never | -2.6 | 0.11 |  |  |  |  |
| HK | Former | 0.1 | 0.02 |  | 0.24 (0.14, 0.34) | 0.1 (-0.4, 0.6) | 0.49 (0.39, 0.58) |
| HK | Current | 3.9 | 0.15 |  |  |  |  |
| XA | Never | 2.2 | 0.33 |  |  |  |  |
| XA | Former | 1.5 | 1.00 |  |  |  |  |
| XA | Current | 3.0 | 0.04 |  | -0.09 (-0.14, -0.05) | 3.0 (2.1, 3.9) | 0.63 (-0.02, 1.27) |
| HAA | Never | 1.5 | 0.28 |  |  |  |  |
| HAA | Former | 1.5 | 0.35 |  |  |  |  |
| HAA | Current | 3.9 | 0.09 |  |  |  |  |
| QA | Never | 0.8 | 2.4E-03 |  | 0.35 (0.26, 0.43) | 0.5 (0.0, 1.0) | 0.69 (0.56, 0.83) |
| QA | Former | 0.1 | 0.02 |  | 0.41 (0.33, 0.49) | 0.3 (-0.2, 0.9) | 0.64 (0.54, 0.74) |
| QA | Current | 0.6 | 3.3E-04 |  | 0.34 (0.28, 0.40) | 0.4 (0.0, 0.8) | 0.56 (0.49, 0.64) |

^1^Adjusted for study center, age, sex, BMI, cotinine, creatinine, CRP, PLP, riboflavin and sample storage time. All continuous variables were log-transformed and then centered on the mean and standardized by dividing by the standard deviation. AA: anthranilic acid; CRP: C-reactive protein; HAA: 3-hydroxyanthranilic acid; HK: 3-hydroxykynurenine; KA: kynurenic acid; KTR: kynurenine/tryptophan ratio; PLP: pyridoxal 5’-phosphate; QA: quinolinic acid; XA: xanthurenic acid.

^2^Results from segmented regression are shown only for biomarkers where Davies test gave a significant breakpoint (p-value <0.05).

^3^Breakpoints are given in SD units relative to the geometric mean of KTR.

Supplementary Figure S7. Neopterin as predictor of tryptophan and kynurenines by Generalized Additive Models (GAM). The models contained cohort, age, sex, BMI, neopterin, CRP, creatinine, cotinine, PLP, riboflavin and sample storage time as predictors. All continuous variables were log-transformed and then centered on the mean and standardized by dividing by the standard deviation. AA: anthranilic acid; CRP: C-reactive protein; HAA: 3-hydroxyanthranilic acid; HK: 3-hydroxykynurenine; KA: kynurenic acid; KTR: kynurenine/tryptophan ratio; Kyn: kynurenine; PLP: pyridoxal 5’-phosphate; Trp: tryptophan; QA: quinolinic acid; XA: xanthurenic acid.

Supplementary Table S7. Relations of neopterin with tryptophan, kynurenines and KTR by Davies test and segmented regression^1^

|  | Davies test | |  | Segmented regression^2^ | | |
| --- | --- | --- | --- | --- | --- | --- |
|  | Breakpoint^3^ | p-value |  | Lower segment | Breakpoint^3^ | Upper segment |
|  |  |  |  | Slope (95% CI) |  | Slope (95% CI) |
| KTR | 0.6 | 2.3E-20 |  | 0.36 (0.32, 0.40) | 0.7 (0.5, 0.9) | 0.80 (0.71, 0.89) |
| Trp | 1.3 | 7.4E-03 |  | -0.08 (-0.13, -0.04) | 0.9 (0.4, 1.4) | -0.31 (-0.43, -0.18) |
| Kyn | -0.1 | 2.0E-08 |  | 0.26 (0.21, 0.31) | 0.3 (0.0, 0.6) | 0.52 (0.45, 0.58) |
| KA | 0.6 | 0.19 |  |  |  |  |
| AA | 0.6 | 7.0E-04 |  | 0.14 (0.09, 0.19) | 0.5 (0.1, 1.0) | 0.35 (0.26, 0.44) |
| HK | -0.1 | 0.01 |  | 0.21(0.15, 0.26) | 0.2 (-0.3, 0.8) | 0.36 (0.29, 0.42) |
| XA | -2.2 | 0.01 |  | 0.00 (-0.04, 0.04) | 1.5 (0.8, 2.2) | -0.25 (-0.50, -0.01) |
| HAA | 3.5 | 0.19 |  |  |  |  |
| QA | -0.1 | 3.2E-18 |  | 0.28 (0.24, 0.33) | 0.2 (0.0, 0.4) | 0.63 (0.57, 0.69) |

^1^Adjusted for study center, age, sex, BMI, cotinine, creatinine, CRP, PLP, riboflavin and sample storage time. All continuous variables were log-transformed and then centered on the mean and standardized by dividing by the standard deviation. AA: anthranilic acid; CRP: C-reactive protein; HAA: 3-hydroxyanthranilic acid; HK: 3-hydroxykynurenine; KA: kynurenic acid; KTR: kynurenine/tryptophan ratio; Kyn: kynurenine; PLP: pyridoxal 5’-phosphate; Trp: tryptophan; QA: quinolinic acid; XA: xanthurenic acid.

^2^Results from segmented regression are shown only for biomarkers where Davies test gave a significant breakpoint (p-value <0.05).

^3^Breakpoints are given in SD units relative to the geometric mean of neopterin.

Supplementary Table S8. Relations of neopterin with tryptophan, kynurenines and KTR by Davies test and segmented regression by age group^1^

|  |  | Davies test | |  | Segmented regression^2^ | | |
| --- | --- | --- | --- | --- | --- | --- | --- |
|  | Age | Breakpoint^3^ | p-value |  | Lower segment | Breakpoint^3^ | Upper segment |
|  | group |  |  |  | Slope 1 (95% CI) |  | Slope 2 (95% CI) |
| KTR | <median | 1.3 | 2.7E-09 |  | 0.36 (0.30, 0.42) | 1.1 (0.8, 1.3) | 0.97 (0.79, 1.16) |
| KTR | >median | -0.2 | 3.3E-06 |  | 0.32 (0.24, 0.40) | -0.1 (-0.4, 0.3) | 0.64 (0.56, 0.71) |
| Trp | <median | 2.7 | 0.11 |  |  |  |  |
| Trp | >median | 0.5 | 0.12 |  |  |  |  |
| Kyn | <median | 0.6 | 3.4E-06 |  | 0.25 (0.19, 0.31) | 0.9 (0.6, 1.2) | 0.68 (0.54, 0.83) |
| Kyn | >median | -0.2 | 9.8E-03 |  | 0.21 (0.10, 0.31) | -0.4 (-0.9, 0.0) | 0.46 (0.39, 0.52) |
| KA | <median | -1.4 | 0.37 |  |  |  |  |
| KA | >median | -0.9 | 0.07 |  |  |  |  |
| AA | <median | 1.3 | 0.28 |  |  |  |  |
| AA | >median | -0.2 | 0.03 |  | 0.10 (-0.02, 0.23) | -0.3 (-0.9, 0.2) | 0.34 (0.26, 0.42) |
| HK | <median | 0.6 | 0.10 |  |  |  |  |
| HK | >median | -0.9 | 0.04 |  | 0.07 (-0.09, 0.22) | -0.7 (-1.2, -0.2) | 0.32 (0.26, 0.38) |
| XA | <median | -1.4 | 0.56 |  |  |  |  |
| XA | >median | 2.6 | 0.54 |  |  |  |  |
| HAA | <median | -1.4 | 0.58 |  |  |  |  |
| HAA | >median | -0.9 | 0.49 |  |  |  |  |
| QA | <median | 1.3 | 1.1E-09 |  | 0.30 (0.25, 0.36) | 1.1 (0.8, 1.3) | 0.89 (0.71, 1.06) |
| QA | >median | -0.9 | 3.4E-05 |  | 0.22 (0.10, 0.33) | -0.6 (-0.9, -0.2) | 0.54 (0.49, 0.60) |

^1^Adjusted for study center, age, sex, BMI, cotinine, creatinine, CRP, PLP, riboflavin and sample storage time. All continuous variables were log-transformed and then centered on the mean and standardized by dividing by the standard deviation. AA: anthranilic acid; CRP: C-reactive protein; HAA: 3-hydroxyanthranilic acid; HK: 3-hydroxykynurenine; KA: kynurenic acid; KTR: kynurenine/tryptophan ratio; Kyn: kynurenine; PLP: pyridoxal 5’-phosphate; Trp: tryptophan; QA: quinolinic acid; XA: xanthurenic acid.

^2^Results from segmented regression are shown only for biomarkers where Davies test gave a significant breakpoint (p-value <0.05).

^3^Breakpoints are given in SD units relative to the geometric mean of neopterin.

Supplementary Table S9. Relations of neopterin with tryptophan, kynurenines and KTR by Davies test and segmented regression by smoking category^1^

|  |  | Davies test | |  | Segmented regression | | |
| --- | --- | --- | --- | --- | --- | --- | --- |
|  | Smoking | Breakpoint^3^ | p-value |  | Lower segment | Breakpoint^3^ | Upper segment |
|  | category |  |  |  | Slope 1 (95% CI) |  | Slope 2 (95% CI) |
| KTR | Never | 0.7 | 0.01 |  | 0.31 (0.20, 0.41) | 0.3 (-0.2, 0.8) | 0.67 (0.50, 0.83) |
| KTR | Former | 0.4 | 1.1E-05 |  | 0.38 (0.30, 0.46) | 0.5 (0.2, 0.9) | 0.82 (0.67, 0.97) |
| KTR | Current | 0.8 | 9.8E-07 |  | 0.34 (0.28, 0.40) | 0.6 (0.3, 0.9) | 0.73 (0.61, 0.85) |
| Trp | Never | -0.7 | 0.28 |  |  |  |  |
| Trp | Former | 1.0 | 0.18 |  |  |  |  |
| Trp | Current | 1.5 | 0.32 |  |  |  |  |
| Kyn | Never | 0.0 | 0.45 |  |  |  |  |
| Kyn | Former | -0.2 | 0.02 |  | 0.28 (0.18, 0.38) | 0.2 (-0.3, 0.7) | 0.55 (0.44, 0.67) |
| Kyn | Current | 0.1 | 3.9E-03 |  | 0.26 (0.19, 0.33) | 0.4 (-0.1, 0.8) | 0.52 (0.42, 0.61) |
| KA | Never | -2.1 | 0.85 |  |  |  |  |
| KA | Former | 2.9 | 0.45 |  |  |  |  |
| KA | Current | 0.8 | 0.33 |  |  |  |  |
| AA | Never | 0.7 | 1.00 |  |  |  |  |
| AA | Former | -0.2 | 0.10 |  |  |  |  |
| AA | Current | 0.8 | 5.8E-03 |  | 0.09 (0.02, 0.16) | 0.7 (0.3, 1.2) | 0.42 (0.26, 0.57) |
| HK | Never | -2.1 | 1.00 |  |  |  |  |
| HK | Former | 0.4 | 0.35 |  |  |  |  |
| HK | Current | 0.1 | 0.27 |  |  |  |  |
| XA | Never | 2.1 | 0.12 |  |  |  |  |
| XA | Former | 2.9 | 0.01 |  | -0.04 (-0.12, 0.04) | 1.2 (0.2, 2.2) | -0.30 (-0.66, 0.05) |
| XA | Current | -2.0 | 1.00 |  |  |  |  |
| HAA | Never | -2.8 | 0.57 |  |  |  |  |
| HAA | Former | 2.3 | 0.93 |  |  |  |  |
| HAA | Current | -2.0 | 0.97 |  |  |  |  |
| QA | Never | 0.7 | 2.8E-03 |  | 0.33 (0.25, 0.42) | 0.6 (0.2, 1.0) | 0.75 (0.56, 0.94) |
| QA | Former | -0.2 | 3.4E-05 |  | 0.24 (0.11, 0.36) | -0.3 (-0.6, 0.1) | 0.62 (0.53, 0.70) |
| QA | Current | 0.1 | 3.8E-07 |  | 0.28 (0.22, 0.35) | 0.4 (0.1, 0.7) | 0.65 (0.56, 0.74) |

^1^Adjusted for study center, age, sex, BMI, cotinine, creatinine, CRP, PLP, riboflavin and sample storage time. All continuous variables were log-transformed and then centered on the mean and standardized by dividing by the standard deviation. AA: anthranilic acid; CRP: C-reactive protein; HAA: 3-hydroxyanthranilic acid; HK: 3-hydroxykynurenine; KA: kynurenic acid; KTR: kynurenine/tryptophan ratio; Kyn: kynurenine; PLP: pyridoxal 5’-phosphate; Trp: tryptophan; QA: quinolinic acid; XA: xanthurenic acid.

^2^Results from segmented regression are shown only for biomarkers where Davies test gave a significant breakpoint (p-value <0.05).

^3^Breakpoints are given in SD units relative to the geometric mean of neopterin.

Supplementary Figure S8. CRP as predictor of tryptophan and kynurenines by Generalized Additive Models (GAM). The models contained cohort, age, sex, BMI, neopterin, CRP, creatinine, cotinine, PLP, riboflavin and sample storage time as predictors. All continuous variables were log-transformed and then centered on the mean and standardized by dividing by the standard deviation. AA: anthranilic acid; CRP: C-reactive protein; HAA: 3-hydroxyanthranilic acid; HK: 3-hydroxykynurenine; KA: kynurenic acid; KTR: kynurenine/tryptophan ratio; Kyn: kyurenine; PLP: pyridoxal 5’-phosphate; Trp: tryptophan; QA: quinolinic acid; XA: xanthurenic acid.

Supplementary Table S10. Relations of CRP with tryptophan, kynurenines and KTR by Davies test and segmented regression^1^

|  | Davies test | |  | Segmented regression^2^ | | |
| --- | --- | --- | --- | --- | --- | --- |
|  | Breakpoint^3^ | p-value |  | Lower segment | Breakpoint^3^ | Upper segment |
|  |  |  |  | Slope (95% CI) |  | Slope (95% CI) |
| KTR | -1.2 | 0.52 |  |  |  |  |
| Trp | 0.8 | 9.3E-03 |  | 0.04 (0.002, 0.07) | 1.1 (0.7, 1.6) | -0.24 (-0.42, -0.06) |
| Kyn | 0.8 | 0.06 |  |  |  |  |
| KA | 0.1 | 9.3E-04 |  | 0.06 (0.01, 0.10) | 0.5 (0.1, 0.9) | -0.16 (-0.24, -0.07) |
| AA | -3.2 | 0.62 |  |  |  |  |
| HK | 2.8 | 0.14 |  |  |  |  |
| XA | 0.8 | 0.014 |  | 0.04 (-0.008, 0.09) | 0.5 (0.0, 1.0) | -0.14 (-0.23, -0.04) |
| HAA | -2.6 | 0.54 |  |  |  |  |
| QA | 1.5 | 0.93 |  |  |  |  |

^1^Adjusted for study center, age, sex, BMI, cotinine, creatinine, CRP, PLP, riboflavin and sample storage time. All continuous variables were log-transformed and then centered on the mean and standardized by dividing by the standard deviation. AA: anthranilic acid; CRP: C-reactive protein; HAA: 3-hydroxyanthranilic acid; HK: 3-hydroxykynurenine; KA: kynurenic acid; KTR: kynurenine/tryptophan ratio; Kyn: kynurenine; PLP: pyridoxal 5’-phosphate; Trp: tryptophan; QA: quinolinic acid; XA: xanthurenic acid.

^2^Results from segmented regression are shown only for biomarkers where Davies test gave a significant breakpoint (p-value <0.05).

^3^Breakpoints are given in SD units relative to the geometric mean of CRP.

Supplementary Table S11. Relations of CRP with tryptophan, kynurenines and KTR by Davies test and segmented regression by age group^1^

|  |  | Davies test | |  | Segmented regression^2^ | | |
| --- | --- | --- | --- | --- | --- | --- | --- |
|  | Age | Breakpoint^3^ | p-value |  | Lower segment | Breakpoint^3^ | Upper segment |
|  | group |  |  |  | Slope (95% CI) |  | Slope (95% CI) |
| KTR | <median | -1.3 | 0.99 |  |  |  |  |
| KTR | >median | -3.1 | 0.08 |  |  |  |  |
| Trp | <median | 1.3 | 0.35 |  |  |  |  |
| Trp | >median | 0.2 | 0.05 |  | 0.09 (0.01, 0.16) | 0.3 (-0.3, 0.9) | -0.13 (-0.24, -0.01) |
| Kyn | <median | 1.3 | 0.28 |  |  |  |  |
| Kyn | >median | 0.2 | 0.31 |  |  |  |  |
| KA | <median | 0.7 | 0.16 |  |  |  |  |
| KA | >median | 0.2 | 0.08 |  |  |  |  |
| AA | <median | -0.6 | 0.67 |  |  |  |  |
| AA | >median | -1.8 | 1.00 |  |  |  |  |
| HK | <median | 2.6 | 2.9E-04 |  | -0.05 (-0.17, 0.07) | -0.5 (-1.2, 0.3) | 0.11 (0.05, 0.17) |
| HK | >median | 2.8 | 0.25 |  |  |  |  |
| XA | <median | 2.6 | 5.5E-03 |  | 0.65 (0.02, 1.28) | -1.8 (-2.3, -1.3) | -0.04 (-0.09, 0.01) |
| XA | >median | 0.8 | 0.03 |  | 0.03 (-0.03, 0.09) | 0.9 (0.4, 1.4) | -0.29 (-0.49, -0.09) |
| HAA | <median | 2.6 | 0.78 |  |  |  |  |
| HAA | >median | 2.8 | 1.00 |  |  |  |  |
| QA | <median | 1.9 | 1.00 |  |  |  |  |
| QA | >median | -0.5 | 1.00 |  |  |  |  |

^1^Adjusted for study center, age, sex, BMI, cotinine, creatinine, CRP, PLP, riboflavin and sample storage time. All continuous variables were log-transformed and then centered on the mean and standardized by dividing by the standard deviation. AA: anthranilic acid; CRP: C-reactive protein; HAA: 3-hydroxyanthranilic acid; HK: 3-hydroxykynurenine; KA: kynurenic acid; KTR: kynurenine/tryptophan ratio; Kyn: kynurenine; PLP: pyridoxal 5’-phosphate; Trp: tryptophan; QA: quinolinic acid; XA: xanthurenic acid.

^2^Results from segmented regression are shown only for biomarkers where Davies test gave a significant breakpoint (p-value <0.05).

^3^Breakpoints are given in SD units relative to the geometric mean of CRP.

Supplementary Table S12. Relations of CRP with tryptophan, kynurenines and KTR by Davies test and segmented regression by smoking category^1^

|  |  | Davies test | |  | Segmented regression^2^ | | |
| --- | --- | --- | --- | --- | --- | --- | --- |
|  | Smoking | Breakpoint^3^ | p-value |  | Lower segment | Breakpoint^3^ | Upper segment |
|  | category |  |  |  | Slope 1 (95% CI) |  | Slope 2 (95% CI) |
| KTR | Never | -0.5 | 0.97 |  |  |  |  |
| KTR | Former | -1.5 | 0.31 |  |  |  |  |
| KTR | Current | 2.6 | 1.00 |  |  |  |  |
| Trp | Never | 0.1 | 0.32 |  |  |  |  |
| Trp | Former | 0.9 | 0.31 |  |  |  |  |
| Trp | Current | 1.3 | 0.31 |  |  |  |  |
| Kyn | Never | -0.5 | 0.19 |  |  |  |  |
| Kyn | Former | 2.8 | 0.33 |  |  |  |  |
| Kyn | Current | 0.7 | 0.57 |  |  |  |  |
| KA | Never | 0.1 | 0.37 |  |  |  |  |
| KA | Former | 1.6 | 0.03 |  | 0.04 (-0.02, 0.09) | 1.9 (1.5, 2.3) | -1.20 (-2.26, -0.14) |
| KA | Current | 0.7 | 0.07 |  |  |  |  |
| AA | Never | -2.9 | 1.00 |  |  |  |  |
| AA | Former | 2.8 | 0.16 |  |  |  |  |
| AA | Current | -3.1 | 0.85 |  |  |  |  |
| HK | Never | 2.6 | 0.16 |  |  |  |  |
| HK | Former | 2.8 | 0.41 |  |  |  |  |
| HK | Current | -2.5 | 0.24 |  |  |  |  |
| XA | Never | 2.6 | 0.22 |  |  |  |  |
| XA | Former | 1.6 | 1.4E-03 |  | 0.05 (-0.007, 0.12) | 1.6 (1.3, 1.9) | -1.02 (-1.67, -0.37) |
| XA | Current | -1.2 | 0.15 |  |  |  |  |
| HAA | Never | 2.6 | 0.93 |  |  |  |  |
| HAA | Former | 2.2 | 0.37 |  |  |  |  |
| HAA | Current | -2.5 | 0.31 |  |  |  |  |
| QA | Never | 2.0 | 0.28 |  |  |  |  |
| QA | Former | 2.8 | 1.00 |  |  |  |  |
| QA | Current | 1.9 | 1.00 |  |  |  |  |

^1^Adjusted for study center, age, sex, BMI, cotinine, creatinine, CRP, PLP, riboflavin and sample storage time. All continuous variables were log-transformed and then centered on the mean and standardized by dividing by the standard deviation. AA: anthranilic acid; CRP: C-reactive protein; HAA: 3-hydroxyanthranilic acid; HK: 3-hydroxykynurenine; KA: kynurenic acid; KTR: kynurenine/tryptophan ratio; Kyn: kynurenine; PLP: pyridoxal 5’-phosphate; Trp: tryptophan; QA: quinolinic acid; XA: xanthurenic acid.

^2^Results from segmented regression are shown only for biomarkers where Davies test gave a significant breakpoint (p-value <0.05).

^3^Breakpoints are given in SD units relative to the geometric mean of CRP.

Supplementary Figure S9. Creatinine as predictor of tryptophan and kynurenines by Generalized Additive Models (GAM). The models contained cohort, age, sex, BMI, neopterin, CRP, creatinine, cotinine, PLP, riboflavin and sample storage time as predictors. All continuous variables were log-transformed and then centered on the mean and standardized by dividing by the standard deviation. AA: anthranilic acid; CRP: C-reactive protein; HAA: 3-hydroxyanthranilic acid; HK: 3-hydroxykynurenine; KA: kynurenic acid; KTR: kynurenine/tryptophan ratio; Kyn: kyurenine; PLP: pyridoxal 5’-phosphate; Trp: tryptophan; QA: quinolinic acid; XA: xanthurenic acid.

Supplementary Table S13. Total variance of KTR, tryptophan and kynurenines explained by Generalized Additive Models (GAM) and Relaimpo^1^

| \|  \| % of total variation explained \| \| \| \| \| --- \| --- \| --- \| --- \| --- \| \|  \| GAM^2^ \| GAM^3^ \| Relaimpo^2^ \| Relaimpo^3^ \| \| KTR \|  \| 42.7 \|  \| 41.1 \| \| Trp \|  \| 19.6 \|  \| 18.7 \| \| Kyn \|  \| 42.4 \|  \| 41.6 \| \| KA \| 36.3 \| 32.5 \| 35.8 \| 31.8 \| \| AA \| 19.9 \| 18.3 \| 19.6 \| 17.6 \| \| HK \| 36.2 \| 34.0 \| 36.1 \| 33.7 \| \| XA \| 15.4 \| 15.0 \| 14.9 \| 14.2 \| \| HAA \| 25.4 \| 26.3 \| 25.3 \| 26.3 \| \| QA \| 56.2 \| 51.3 \| 55.1 \| 49.6 \| |  |
| --- | --- | --- | --- | --- | --- | --- | --- | --- | --- | --- | --- | --- | --- | --- | --- | --- | --- | --- | --- | --- | --- | --- | --- | --- | --- | --- | --- | --- | --- | --- | --- | --- | --- | --- | --- | --- | --- | --- | --- | --- | --- | --- | --- | --- | --- | --- | --- | --- | --- | --- | --- | --- | --- | --- | --- | --- |

^1^All models included cohort, age, sex, BMI, KTR or neopterin, CRP, creatinine, cotinine, PLP, riboflavin and sample storage time. All continuous variables were log-transformed and then centered on the mean and standardized by dividing by the standard deviation. AA: anthranilic acid; BMI: body mass index; CRP: C-reactive protein; HAA: 3-hydroxyanthranilic acid; HK: 3-hydroxykynurenine; KA: kynurenic acid; KTR: kynurenine/tryptophan; Kyn: kynurenine; PLP: pyridoxal 5’-phosphate; Trp: tryptophan; QA: quinolinic acid; XA: xanthurenic acid.

^2^Results from models that included KTR, but not neopterin as predictor.

^3^Results from models that included neopterin, but not KTR as predictor.
